# A next generation sequencing based approach to identify extracellular vesicle mediated mRNA transfers between cells

**DOI:** 10.1186/s12864-017-4359-1

**Published:** 2017-12-22

**Authors:** Jialiang Yang, Jacob Hagen, Kalyani V. Guntur, Kimaada Allette, Sarah Schuyler, Jyoti Ranjan, Francesca Petralia, Stephane Gesta, Robert Sebra, Milind Mahajan, Bin Zhang, Jun Zhu, Sander Houten, Andrew Kasarskis, Vivek K. Vishnudas, Viatcheslav R. Akmaev, Rangaprasad Sarangarajan, Niven R. Narain, Eric E. Schadt, Carmen A. Argmann, Zhidong Tu

**Affiliations:** 1 Institute of Genomics and Multiscale Biology, Icahn School of Medicine at Mount Sinai, New York, NY 10029 USA; 2 Department of Genetics and Genomic Sciences, Icahn School of Medicine at Mount Sinai, New York, NY 10029 USA; 3 BERG, LLC, Framingham, MA 01701 USA

**Keywords:** Extracellular RNA (exRNA), Exosome, Cell-to-cell communication, RNA-seq, Macrophages, Adipocytes, Bayesian method

## Abstract

**Background:**

Exosomes and other extracellular vesicles (EVs) have emerged as an important mechanism of cell-to-cell communication. However, previous studies either did not fully resolve what genetic materials were shuttled by exosomes or only focused on a specific set of miRNAs and mRNAs. A more systematic method is required to identify the genetic materials that are potentially transferred during cell-to-cell communication through EVs in an unbiased manner.

**Results:**

In this work, we present a novel next generation of sequencing (NGS) based approach to identify EV mediated mRNA exchanges between co-cultured adipocyte and macrophage cells. We performed molecular and genomic profiling and jointly considered data from RNA sequencing (RNA-seq) and genotyping to track the “sequence varying mRNAs” transferred between cells. We identified 8 mRNAs being transferred from macrophages to adipocytes and 21 mRNAs being transferred in the opposite direction. These mRNAs represented biological functions including extracellular matrix, cell adhesion, glycoprotein, and signal peptides.

**Conclusions:**

Our study sheds new light on EV mediated RNA communications between adipocyte and macrophage cells, which may play a significant role in developing insulin resistance in diabetic patients. This work establishes a new method that is applicable to examining genetic material exchanges in many cellular systems and has the potential to be extended to in vivo studies as well.

**Electronic supplementary material:**

The online version of this article (10.1186/s12864-017-4359-1) contains supplementary material, which is available to authorized users.

## Background

Cell-to-cell communication plays a key role in maintaining the integrity of multicellular systems. The most studied cell-to-cell communication mechanisms include chemical or hormone-mediated signaling and direct cell-to-cell contacts. In late 1990’s, exchange of cellular information was also demonstrated to occur via release of intracellular contents packaged in lipid bilayer vesicles called extracellular vesicles (EVs) or exosomes as reviewed by Colombo et al. [1]. Various types of EVs exist and are generally classified according to their sub-cellular origins. Microvesicles (MVs) are EVs formed and released by budding from cells plasma membrane and display a diverse range of sizes (100–1000 nm in diameter). Exosomes, in contrast, are of endosomal origin and are released as a consequence of multivesicular endosomes fusing with the plasma membrane and are generally small in size (30–150 nm). Importantly, EV secretion appears to be conserved throughout evolution and is a characteristic of most cell types including adipocytes, macrophages, hematopoietic, neuronal, fibroblastic, and various tumour cells [2, 3]. Furthermore, these EVs have been found to contain various types of cargo, such as mRNA, microRNA, proteins, lipids, and DNA [4–6], representing diverse ways of mediating cell-to-cell communications. As EVs contain surface molecules that can be recognized by recipient cells, these molecules can be readily shuttled from one cell to another and thereby influence the biological state of the recipient cell in multiple ways [1].

With the potential of EVs as critical mediators of cell-to-cell communication, they have become a key focus of research for numerous pathological settings as biomarkers as well as mediators of disease. A role for EVs has been subsequently identified in various disease settings, including diabetes, cardiovascular disease, inflammation and pain, degenerative brain disorders and cancer, to name a few. For example, Deng et al. demonstrated that exosome-like vesicles obtained from obese mice was sufficient to induce insulin resistance when injected into wild-type C57BL/6 mice [2]. Ibrahim et al. pinpointed exosomes secreted by human cardio sphere-derived cells (CDCs) as critical agents of regeneration of injured heart muscle and cardio protection. They found that the injection of exosomes into injured mouse hearts recapitulated the regenerative and functional effects produced by CDC transplantation, whereas inhibition of exosome production by CDCs blocks these benefits [7]. With respect to cancers, exosomal microRNAs and RNA are in fact being used as diagnostic biomarkers for several cancers such as ovarian cancer [8]. Moreover, exosomes have been adopted as carriers to load MHC class I and class II peptides for vaccinating metastatic melanoma patients [9] and to deliver antitumor microRNA let-7a to treat breast cancer in mice [10]. The readers are referred to recent review articles for detailed roles of exosomes in disease processes [11, 12].

Despite the seemingly extensive characterization of EVs to date, however, limitations exist. First, though several studies have identified many genetic materials within EVs, they were not detailed as necessarily being transported and released into recipient cells [13]. Secondly, there is a lack of estimation of the transferring rate, e.g., the proportion of total genetic materials (in the form of mRNA, miRNA, protein, etc.) being transferred from one cell to another. Mittelbrumn et al. demonstrated the existence of antigen-driven unidirectional miRNA transfer from the T cell to the antigen-presenting cells [14]. However, it is not clear if the unidirectional transfer is true between other cell types. For these reasons, a more systematic method is needed to identify the genetic materials that are potentially transferred during cell-to-cell communication through EVs in an unbiased manner.

In this study, we jointly consider data from RNA sequencing and genotype array to systematically discover mRNA exchanges between two co-cultured cell lines of different genetic backgrounds. We relate those exchanges to potential EV mediated transfer by verifying them with mRNA sequencing of purified EVs. Given the precedent for potential crosstalk between macrophages and adipocytes in adipose tissue under obese conditions leading to insulin resistance [15], we chose to apply our novel methodology to co-cultures of human differentiated adipocytes and macrophages.

## Methods

### Experimental design and data generation

#### Experimental design

**Fig. 1:**
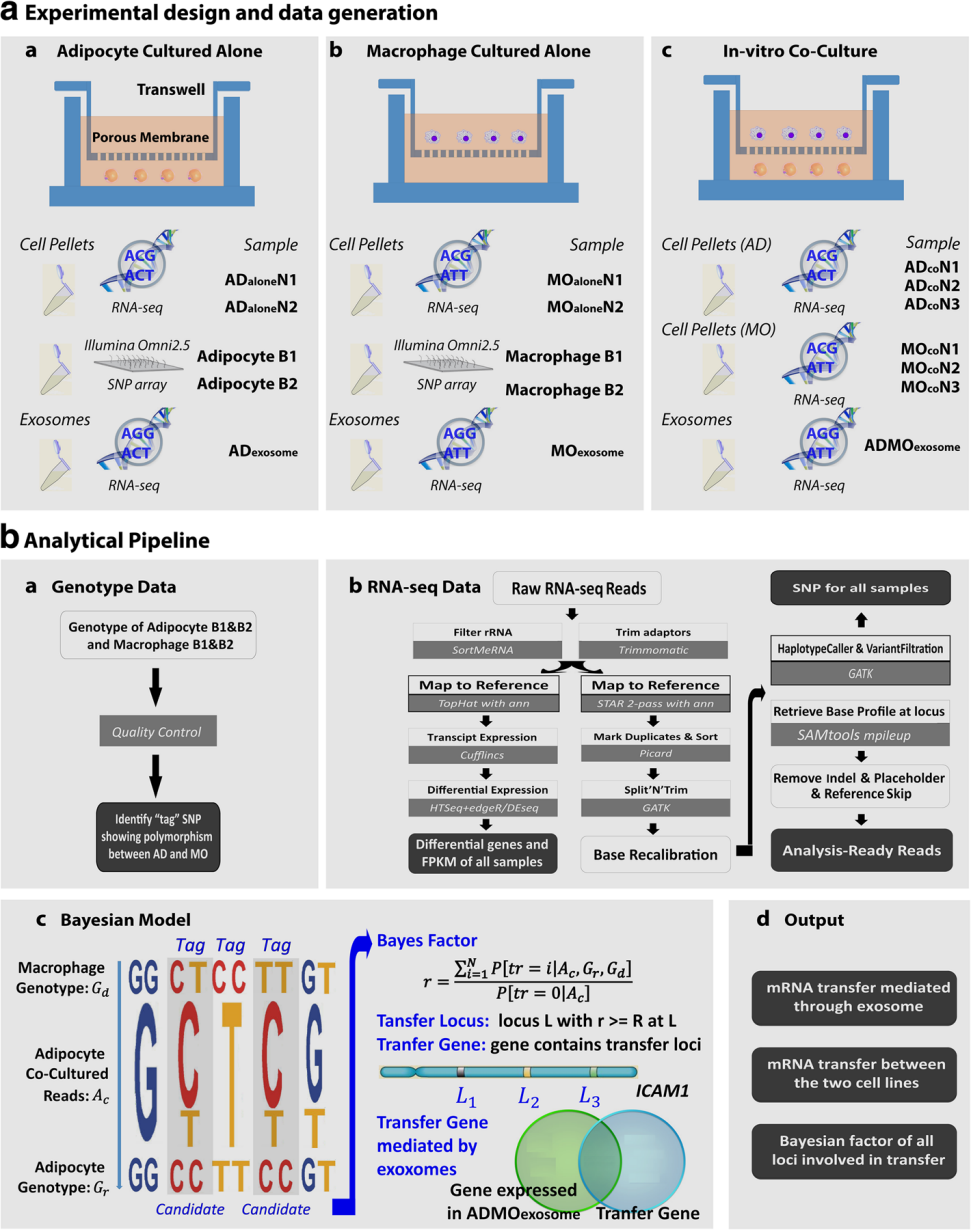
The experimental workflow used to identify mRNA transfers between adipocytes and macrophages in a co-culture system: (**a**) A schema illustrating the experimental design for cell culture and sample collection including (a) adipocyte cells cultured alone, from which two cell pellet samples AD_alone_N1 and AD_alone_N2 were retrieved for RNA-seq, adipocyte B1 and B2 were technical duplicates of genotyping using Illumina Omni2.5 SNP array, and an exosome extraction AD_exosome_ was prepared for RNA-seq; (b) same as (a) but for macrophage cells; (c) co-culture of two cell lines, from which three adipocyte cell pellet samples AD_co_N1-N3, three macrophage cell pellet samples MO_co_N1-N3, and an exosome sample ADMO_exosome_ were profiled by RNA-seq. **b** The analytical pipeline including the pre-processing of (a) genotype data and (b) RNA-seq data, (c) the Bayesian model to call mRNA transfers between two cell lines, and (d) the final output of this pipeline

Our main experiment was performed on an in vitro co-culture cellular system, in which two types of human cells, namely, the adipocytes and macrophages were cultured in transwell plates with porous membrane inserts (pore size of 0.4 μm) to prevent them from being mixed together (Corning, Inc. Costar, NY, USA, see Additional file 1). The porous membrane allows small particles (size less than 0.4 μm) to pass through, making EV mediated mRNA exchanges between the two cell lines possible, which has been demonstrated by Garcia et al. [16] and Zheng et al. [17] (Fig. 1a and Additional file 1: Figure S1). The two cell lines were derived from donors with no known familial relationships. Therefore, a large number of single nucleotide polymorphic (SNP) markers between the two cell lines were expected which would allow us to easily distinguish cell origins. With this experimental setting, we planned to address: if there are mRNA exchanges detectable between the two cell lines; what the genes being transferred and their functions are; and finally if any transferred mRNAs are likely mediated by EVs in the media.

#### Data generation

We performed genotype profiling using the Illumina HumanOmni2.5Exome-8 BeadChip for the two cell lines with two technical replicates for each cell line (Adipocyte (AD) B1/B2 and Macrophage (MO) B1/B2). We also performed RNA-seq profiling for both single cultured and co-cultured cells. For single cultured cells, we generated RNA-seq data from two replicate cell pellet samples (ADaloneN1/N2, and MOaloneN1/N2). For the co-culture cells, we obtained the RNA-seq data from triplicate cell pellet samples in the adipocyte layer (ADcoN1-N3) and triplicate cell pellet samples in the macrophage layer (MOcoN1-N3). EVs were isolated using sequential ultracentrifugation and western blotting demonstrated enrichment for CD9 expression, a marker of endosomal origin. Considering the precedence set in the field, where a mixture of exosomes and MV is likely, we are being conservative and referring to what we isolated in the media broadly as EVs also referred to as ‘exosome-like vesicles’. We performed RNA-sequencing on isolated EVs from media of single cultured and co-cultured cells and profiled the data of ADexosome, MOexosome, and ADMOexosome (Fig. 1a). The readers are referred to Additional file 1 for details regarding genotyping and RNA sequencing on both cells and EVs.

### Data processing and quality control

#### NGS data processing

We first conducted a multiple-step quality control (QC). Briefly, we filtered out ribosomal RNAs (rRNAs) using SortMeRNA [18] and trimmed Illumina adaptors using Trimmomatic [19] on raw pair-ended RNA-seq reads (of length 100 bps) for each sample. 11~90 M pairs of reads were obtained after these two QC steps for all samples except ADexosome which generated much fewer reads (528 K pairs reads). These reads were then mapped to reference human genome (hg38) using an annotation file (GENCODE V21) by STAR (2.4.0.1) [20]. The mapping rates were over 90% for all cell samples and over 80% for exosomes (Additional file 1: Table S1). The uniquely mapped reads were further processed by several steps, such as marking duplicates, Split’N’Trim, reassigning mapping quality, base call recalibration, etc., similar to the protocol used in [21] (Fig. 1b). SAMtools mpileup [22] was used to retrieve the base profile at each locus (see Additional file 1 for more details). We disregarded mapped indel, placeholder, and reference skip bases and only considered remaining bases for our analysis.

We adopted TopHat, HTSeq, and edgeR/DEseq protocols [23] to identify differentially expressed genes between single-cultured and co-cultured samples. Cufflinks protocol [24] was used to quantify gene expression, and GATK [21] to call variants in each sample. For cross sample quality control, we applied Principal Component Analysis (PCA) and the results based on the first two PCs for gene expressions were plotted in Additional file 1: Figure S2. We also showed the consistency of genotypes on common SNPs across 10 cell samples and 2 exosome samples in Additional file 1: Figure S3. Both gene expression levels and variants were consistent with sample annotations, suggesting no sample mislabelling occurred. To maximize the power of detecting mRNA transfer, we also merged RNA-seq reads from the same cell lines and denoted them as ADalone, ADco, MOalone, and MOco, respectively.

### Analytical procedures for identifying transferred mRNAs

At a SNP locus, five typical scenarios of nucleotide base composition exist as illustrated in Fig. 1b (c). We define a SNP as a “tag” SNP if it satisfies the following two criteria: (1) shows a polymorphism between the two cell lines; and (2) it has a homozygous genotype in the recipient cell. Tag SNPs are informative to us to infer if donor cells have provided some copies of their mRNAs to the recipient cells using RNA-seq data. Two candidate tag SNPs are highlighted by grey background shading. Using the first highlighted case as an example, the genotype at this locus is “CT” for macrophages and “CC” for adipocytes. For adipocytes under co-culture condition, the mapped reads data contain both “C” and “T” bases, in which the “T” bases may come from macrophages during in vitro co-culture. Alternatively, it is possible that the “T”s in the adipocytes may originate from other mechanisms such as sequencing error, mapping error, and RNA editing. We developed a Bayesian model to distinguish mRNA transfers from the alternative mechanisms. Considering adipocytes co-cultured with macrophages, for a given locus, the Bayesian model takes the following as inputs: (1) the genotype information of donor (macrophage) and recipient (adipocyte) cell lines; (2) the counts of the nucleotides at the locus, and (3) base qualities of the reads mapped to the locus.

Specifically as shown in Additional file 1: Figure S4, we denote the mapped RNA-seq read data at a particular genome position from four profiles by *A*_*I*_, *A*_*C*_, *M*_*I*_, and *M*_*C*_, corresponding to adipocyte alone, adipocyte co-cultured, macrophage alone, and macrophage co-cultured cells, respectively. Let *G*_*d*_ and *G*_*r*_ be the genotypes of donor cells and receptor cell respectively. When examining the sequence data of adipocyte co-cultured with macrophage, *G*_*d*_ would be the genotype of macrophage (donor cell) and *G*_*r*_ would be the genotype of adipocyte (receptor cell). We assume that the read depth of *A*_*C*_ is *N* and denote *t*_*r*_ (0 ≤ *t*_*r*_ ≤ *N*) as the number of reads in *A*_*C*_ that were transferred from donor cells. Obviously, a genetic material transfer happens at the position of consideration if *t*_*r*_ ≥ 1.

We frame the question as a Bayesian inference. We calculate the Bayes factor *r* to measure the confidence ratio of observing the data under two hypotheses, i.e., there exists at least one transfer vs. there is no transfer (null hypothesis).

$$ r=\frac{\sum \limits_{i=1}^NP\left[{t}_r=i,{A}_C,{G}_r,{G}_d\right]}{P\left[{t}_r=0|{A}_C\right]} $$

We reject null hypothesis, and claim a positive genetic material transfer if *r*is greater than a predefined threshold. We assume that the true genotype of the donor and receptor cells are known, i.e., *G*_*r*_and *G*_*d*_are predefined constants (based on genotype array data). We next calculate *P*[*t*_*r*_ = *i*| *A*_*C*_] with 0 ≤ *i* ≤ *N*, that is, the posterior probability of exactly *i* nucleotides being transferred given *A*_*C*_.

To calculate *P*[*t*_*r*_ = *i*| *A*_*C*_, *G*_*r*_, *G*_*d*_]*,* based on Bayesian rule,

1 $$ {\displaystyle \begin{array}{l}P\left[{t}_r=i|{A}_C,{G}_r,{G}_d\right]=\frac{P\left[{A}_C|{t}_r=i,{G}_r,{G}_d\right]P\left[{t}_r=i|{G}_r,{G}_d\right]}{P\left[{A}_C,{G}_r,{G}_d\right]}\\ {}\kern7.75em =\frac{P\left[{A}_C|{t}_r=i,{G}_r,{G}_d\right]P\left[{t}_r=i\right]P\left[{G}_r,{G}_d\right]}{P\left[{A}_C,{G}_r,{G}_d\right]}\kern1.25em \end{array}} $$

where the denominator *P*[*A*_*C*_, *G*_*r*_, *G*_*d*_] is a constant and is often omitted in Bayesian calculation. The *P*[*t*_*r*_ = *i*, *G*_*r*_, *G*_*d*_] = *P*[*t*_*r*_ = *i*]*P*[*G*_*r*_, *G*_*d*_] holds as we assume that the number of reads being transferred is independent of the genotype of donor or acceptor cells.

It is of note that we derive *G*_*r*_ and *G*_*d*_ based on genotype array data, but given we also have the RNA-seq data, an alternative approach is to estimate *P*[*G*_*r*_] and *P*[*G*_*d*_] by *P*[*G*_*r*_| *A*_*I*_] and *P*[*G*_*d*_| *M*_*I*_], which could be calculated in the same way as McKenna et al. [25]. This alternative approach can be useful when genotype information is not available.

*P*[*t*_*r*_ = *i*] is the prior “belief” that *i* reads were from transfer at the position under consideration. Without much prior knowledge of transfer, we assume a uniform prior, so that, $$ P\left[{t}_r=i\right]=\left\{\begin{array}{l}\frac{1}{2}, if\ i=0\\ {}\frac{1}{2N}, if\ i\ne 0\end{array}\right. $$. That is to assume equal probabilities of having genetic material transfer and not having a transfer, and further assume that the probability of transferring *i* nucleotides to be equal for any *i* with 1 ≤ *i* ≤ *N*.

To calculate*P*[*A*_*C*_| *t*_*r*_ = *i*, *G*_*r*_, *G*_*d*_], we assume that reads are independent to each other, thus $P\left[{A}_C|{t}_r=i,{G}_r,{G}_d\right]=\prod \limits_{j=1}^NP\left[{b}_j|{t}_r=i,{G}_r,{G}_d\right]$, where *b*_*j*_ is the nucleotide observed for read *j*. For *N* reads in *A*_*C*_, if *i* of them were from transfer, each read has a probability of $\frac{i}{N}$ for coming from a transfer and a probability of $\frac{N-i}{N}$ for not from transfer. Thus, $P\left[{b}_j|{t}_r=i,{G}_r,{G}_d\right]=\frac{i}{N}P\left[{b}_j|{G}_d\right]+\frac{N-i}{N}P\left[{b}_j|{G}_r\right]$. In addition, for those transferred reads, they are from either one of the parental chromosomes in the donor cell. By assuming that the probability of transferring from maternal chromosome is equal to the probability from paternal chromosome, (i.e., we ignore the situation of allele specific expression), we get$P\left[{b}_j|{G}_d\right]=P\left[{b}_j|\left\{{A}_1,{A}_2\right\}\right]=\frac{1}{2}P\left[{b}_j|{A}_1\right]+\frac{1}{2}P\left[{b}_j|{A}_2\right]$, where the genotype *G*_*d*_ = {*A*_1_, *A*_2_} is decomposed into its two alleles. The probability of observing a base given an allele is

$$ P\left[{b}_j|A\right]=\left\{\begin{array}{l}1-{10}^{\frac{-Q}{10}}, if\ {b}_j=A\ \\ {}\frac{1}{3}{10}^{\frac{-Q}{10}}, if\ {b}_j\ne A\ \end{array}\right. $$

Where *Q* is the *phred* scaled recalibrated quality score at the base. Similarly, we can calculate*P*[*b*_*j*_| *G*_*r*_].

**Fig. 2:**
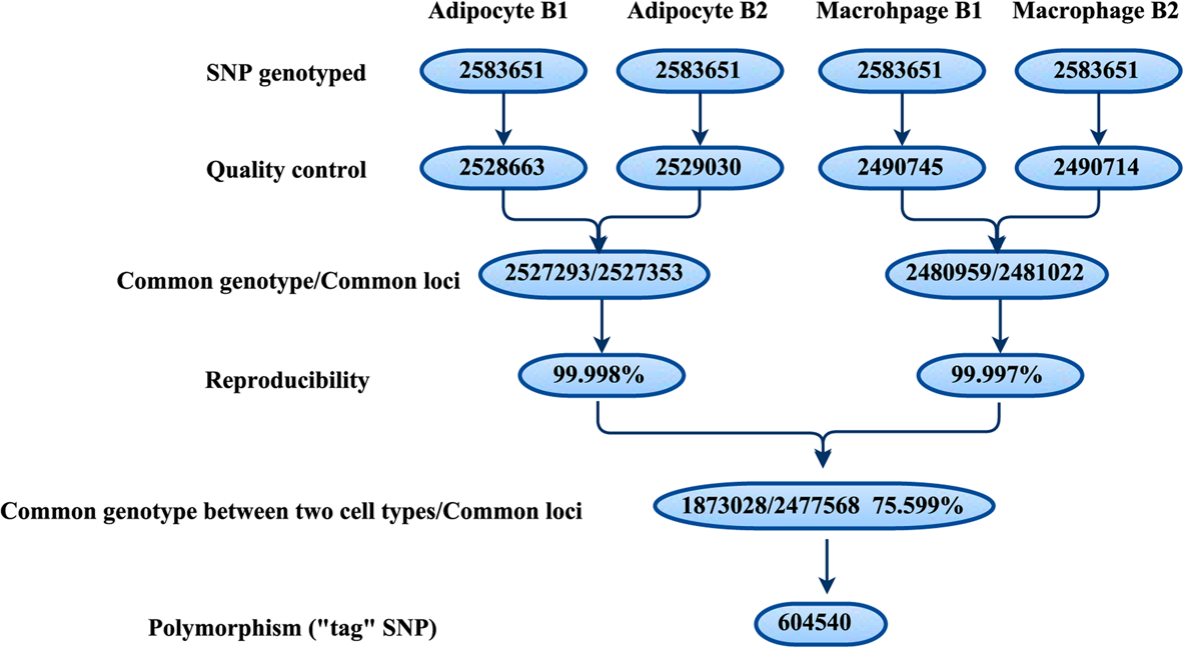
Genotyping quality control and comparison between adipocyte and macrophage cell lines: There are two cell lines each with two technical replicates for their genotyping profiling (Adipocyte B1/B2 and Macrophage B1/B2). The number in each eclipse denotes the number of SNPs kept at that step for the corresponding sample. There are 604,540 “tag” SNPs showing polymorphisms between the two cell lines

Based on the method described above, we calculate the Bayesian factor for each locus. We call genetic material transfer occurred at a locus if the Bayes factor is greater than a predefined threshold *β* (*β* = 20). We performed the analysis on all ~605,000 tag SNPs (Fig. 2) with read depth equal to or greater than 10 in the co-cultured samples. Finally, we evaluated the possibility of mRNA transfers being mediated through exosomes by cross-referencing to the identity of mRNAs in the EVs (Fig. 1b).

It is of note that a very small fraction of loci showed inconsistency between the genotyping data and the RNA-seq reads profile. For example, at chr1:145,746,933, the genotype of adipocyte cell line is “GG”, however all the 56 reads and all the 38 reads mapped to this locus are “C”s in ADaloneN1 and ADaloneN2, respectively (Additional file 2: Dataset S1). Such inconsistent loci were likely caused by genotyping errors, alignment errors, errors in the reference genome, etc., and could be problematic for our down-stream analysis. Therefore, we filtered out genotypes if in the single cultured recipient cells, the proportion of reads different from genotype was larger than a predefined threshold *γ*. With *γ* = 0.005, 9861 (2.26%) and 14,028 (3.22%) loci were filtered out for adipocytes and macrophages, respectively (Additional file 2: Dataset S1).

### Estimating FDR

We calculated the Bayesian factors for triplicate co-cultured samples (i.e., ADcoN1-N3: adipocytes co-cultured with macrophages, and MOcoN1-N3: macrophages co-cultured with adipocytes) and ranked target loci accordingly (loci with larger Bayesian factor rank to the top) in each sample. We then adopted a robust rank aggregation method [26] to identify loci that were ranked consistently better than expected under null hypothesis of uncorrelated inputs and assigned a significance score (*p*-value) to each locus. Finally, we adjusted the *p*-values for multiple comparisons to estimate the false discovery rate (FDR).

## Results

### Identify mRNA transfers potentially mediated by EVs in co-cultured adipocytes and macrophages

We explored mRNA transfers in two directions, i.e., from macrophage to adipocyte and from adipocyte to macrophage, respectively.

#### From macrophage to adipocyte

Ten thousand ninety-five loci survived the filtering steps in the analytical procedures. We further required the potential transfer loci to (1) have Bayesian factor larger than or equal to 20 in at least one sample (i.e., ADcoN1-N3), and (2) contain at least 2 reads that are potentially transferred from donor cells (e.g., “T” allele for the example in analytical procedures in ADco (the merged data from ADcoN1-N3)). Three hundred twenty-one loci satisfied such requirements (Additional file 3: Dataset S2). Among these loci, we identified 8 (corresponding to 8 unique genes) that are putative mRNA transfers from macrophage to adipocytes with high confidence (FDR < 0.1, Table 1). All these 8 genes were likely mediated by EVs as they were all expressed in both MOexosome and ADMOexosome (Table 1). It is of note that the number of total transcripts in GENCODE V21 is 60,566, among which 13,697 have FPKM larger than 0 and only 1966 larger than 1 in ADMOexosome RNA expression. The Fisher’s exact test indicates that the overlap between inferred transferred genes and those expressed in ADMOexosome are very significant (*p*-value < 2.2E-16) (Table 2). We also used Integrative Genomics Viewer (IGV) [27] to visually verify the 8 genes and showed the alignment (of MOaloneN1-N2, ADcoN1-N3, and ADaloneN1-N2) at chr19:10,286,547 (*ICAM1*) in Additional file 1: Figure S5. As shown in Additional file 1: Figure S5, the read counts summarized by IGV were consistent with the read counts we calculated and listed in Table 1.

**Table 1:** Top loci identified as transferred from macrophage to adipocyte possibly mediated through macrophage-derived exosomes

**Location**	**Gene** **Symbol**	**AD** **GT** ^**a**^	**MO** **GT**	**Allele** **Type** ^**b**^	**AD**_**co**_ **N1**		**AD**_**co**_ **N2**		**AD**_**co**_ **N3**		**AD**_**alone**_ **Count**	**FDR**	**ADMO**_**exosome**_ **FPKM** ^**d**^	**MO**_**exosome**_ **FPKM**
					**BF** ^**c**^	**Count**	**BF**	**Count**	**BF**	**Count**				
chr19:10,286,547	ICAM1	T/T	C/C	T|C	4.91E + 03	16|2	2.68E + 11	94|7	1.08E + 07	44|4	37	1.96E-06	1.89E-01	7.29E-01
chr2:237,340,987	COL6A3	T/T	T/C	T|C|G	2.03E + 03	2450|11|0	9.67E + 00	2999|8|1	6.95E + 03	1851|8|2	2184|6|1	1.07E-03	1.58E-02	1.13E + 00
chr9:116,153,914	PAPPA	G/G	A/G	G|A|T	6.51E + 04	766|6|0	1.99E + 02	1963|6|4	1.37E + 00	1464|3	572|1|0	2.65E-03	9.61E-03	1.26E-01
chr11:110,127,909	ZC3H12C	G/G	A/G	G|A	1.05E + 17	4|6	1.73E + 11	6|4	NA	NA	2	2.67E-03	4.81E-02	2.08E-01
chr20:58,995,401	CTSZ	C/C	T/T	C|T	5.97E-01	120|1	3.53E + 02	392|3	5.92E + 02	288|3	377	4.13E-03	2.31E-01	2.92E-01
chr18:23,544,981	NPC1	G/G	C/C	G|C|A	1.81E + 00	16|1|0	3.66E + 03	152|3|1	9.73E-01	74|1|0	49	1.29E-02	1.19E + 00	2.12E + 00
chr2:117,918,535	CCDC93	C/C	T/T	C|T	3.57E + 00	48|1	2.34E + 00	53|1	8.53E + 00	27|1	55	4.26E-02	3.57E-01	1.57E + 00
chr21:39,177,540	PSMG1	C/C	T/T	C|T	5.60E-01	27|1	1.30E + 00	72|1	4.95E + 00	36|1	86	9.33E-02	2.08E + 00	8.31E + 00

^a^ADGT (MOGT) denotes the genotype of adipocyte (macrophage) reported by SNP array

^b^AlleleType (Count) denotes all alleles (the number of alleles) mapped at the corresponding location by the RNA-seq data on replicated samples

^c^The Bayesian factor calculated at the specific locus for the corresponding sample

^d^The fpkm of the gene at ADMOexosome as quantified by Cufflinks

**Table 2:** Number of genes involved in the transfer from adipocytes to macrophages, those possibly mediated by exosomes, and their overlap significance

	# transcripts measured in ADMO_exosome_	# Sig Gene^a^	FPKM > 0			FPKM > 1		
			All^b^	overlap	*p*-value^c^	all	overlap	*p*-value
AD to MO	60,566	20	13,697	17	<2.2E-16	1966	5	< 2.2E-16
MO to AD	60,566	8	13,697	8	<2.2E-16	1966	2	< 2.2E-16

^a^The significant gene is defined at FDR < = 0.05

^b^All (overlap) denotes the number of transcripts with FPKM > 0 (and overlapping with significant genes) for ADMOexosome

^c^The p-value is obtained by testing the overlapping significance between “All” and “Sig Gene” using the Fisher’s exact test with all 60,566 transcripts measured as background

A close inspection of the individual genes showed that several of them belonged to the extracellular space (GO:0005615; *PAPPA*, *ICAM1*, *CTSZ* and *COL6A3*) and lysosome (GO:0005764 *NPC1* and *CTSZ*) GO categories. One unifying theme identified from the top three transfers was related to impacting pathways associated with insulin resistance. For example, our top hit *ICAM1* (Intercellular Adhesion Molecule 1) from macrophages to adipocytes is an endothelial- and leukocyte-associated transmembrane protein long known for its importance in stabilizing cell-cell interactions and facilitating leukocyte endothelial transmigration [28]. It has also been demonstrated to associate with insulin resistance and diabetic retinopathy in type 2 diabetes (T2D) mellitus [29, 30]. Interestingly, although ICAM-1 is often annotated as a transmembrane protein, two types of extracellular ICAM-1 have also been detected outside of cells or in serum including a soluble form and a membranous form associated with exosomes.

In addition to inflammatory mediators like ICAM-1, factors related to extracellular matrix (ECM) components of the adipose tissue have recently emerged as important mediators in obesity-related pathogenesis. In particular one of the most abundantly expressed collagens in the adipose tissue forming part of the ECM structure is COL6 and its alpha 3 chain, *COL6A3* has been associated with adipose tissue inflammation and fibrosis. In collagen VI knockout (KO) mouse for example on an *ob/ob* background, adipocytes of the knockout mice were larger than wildtype and blood glucose was normalized suggesting that elements of the ECM restrict expansion of adipocyte during obese insults. Relevance has also been observed in obese humans, with elevated levels of collagen VI being detected as well as significant correlations with macrophage infiltration. Observing mRNA for *COL6A3* in exosomes from macrophages suggests another level by which the ECM may be influenced by the cells of the adipose tissue depots [31, 32].

Finally, rounding out the top three hits, is Pregnancy-associated plasma protein-A (PAPP-A), a secreted metalloproteinase. PAPP-A cleaves insulin-like growth factor binding proteins (IGFBPs), thereby functioning as a growth-promoting enzyme by releasing bioactive IGF in close proximity to the IGF receptor. As PAPP-A has demonstrated to have fat depot-specific expression in humans and mice, and as the IGF signalling is known to regulate various adipose tissue processes in part through influencing the insulin/insulin receptor signalling axis, exosomal derived PAPP-A mRNA may serve as another mechanism whereby PAPP-A elicits its autocrine/paracrine actions [33].

#### From adipocyte to macrophage

Thirty-seven thousand eight hundred fifty-three loci survived the filtering steps in the analytical procedures, among which 599 are potentially involved in the mRNA transfer from adipocyte to macrophage (Additional file 4: Dataset S3). There are 21 high confidence loci with FDR < 0.1, among which 17 are likely mediated by EVs as listed in Table 3. The Fisher’s exact test indicates that the overlap between our inferred genes and those expressed in ADMOexosome are also very significant (*p*-value <2.2E-16) (Table 2). Furthermore, GO ontology analysis of these genes indicated that several of these genes were annotated under the extracellular vesicular exosome (GO:0070062) category (*FCER2*, *UBL3*, *AHNAK*, *DSTN*, *SOD2*, *HSPG2* and *LAMC1*). Similarly, we used Integrative Genomics Viewer [27] to visually check the 17 genes and an example at chr6:75,666,836 (*SENP6*) is shown in Additional file 1: Fig. S6.

**Table 3:** Top loci identified as transferred from adipocyte to macrophage

Location	Gene Symbol	AD GT^a^	MO GT	Allele Type^b^	MO_co_N1		MO_co_N2		MO_co_N3		MO_alone_ Count	FDR	ADMO_exosome_ FPKM^d^
					BF^c^	Count	BF	Count	BF	Count			
chr16:88,718,047	PIEZO1	C/C	T/T	T|C	2.19E + 13	6|5	5.25E + 03	17|2	1.07E + 07	40|4	24	2.78E-06	2.21E-01
chr9:35,657,841	RMRP	A/G	G/G	G|A|T|C|N	1.69E + 32	1917|25|1|0|0	8.25E + 02	20,459|18|6|5|1	1.05E + 03	22,090|19|14|4|0	21,575|10|1|5|0	8.41E-06	1.89E-01
chr1:183,136,570	LAMC1	A/G	G/G	G|A	1.97E + 02	82|2	1.03E + 07	92|4	2.06E + 01	141|2	92	7.65E-05	1.64E-01
chr19:7,692,417	FCER2	T/C	T/T	T|C	8.00E + 00	29|1	1.34E + 02	26|2	1.36E + 03	40|3	49	6.12E-04	6.45E-01
chr6:75,666,836	SENP6	G/G	A/A	A|G|C	6.08E + 05	39|4|0	1.42E + 01	171|2|0	2.60E + 00	224|2|1	131	1.57E-03	1.08E + 00
chr14:77,575,179	SPTLC2	T/C	T/T	T|C	5.67E + 09	8|4	NA	NA	3.72E + 23	23|10	6	1.59E-03	5.10E-01
chr6:159,682,052	SOD2	T/C	C/C	C|T	7.03E + 02	67|2	6.04E + 00	269|2	2.33E + 00	507|2	135	6.30E-03	1.76E + 00
chr4:102,344,186	SLC39A8	A/C	C/C	C|A	1.67E + 04	17|2	1.16E-01	12	1.63E + 03	50|2	13	6.40E-03	1.48E + 00
chr20:17,607,288	DSTN	G/G	A/A	A|G	4.13E + 03	11|2	1.44E + 06	124|5	3.29E-02	297|1	163	9.72E-03	1.33E + 00
chr20:41,180,020	ZHX3	G/G	T/T	T|G	5.58E + 00	11|1	1.49E + 02	27|2	5.96E-01	67|1	30	1.42E-02	5.09E-01
chr13:29,766,448	UBL3	T/C	T/T	T|C	4.14E-01	60|1	1.74E + 00	135|2	6.74E + 00	104|2	107	1.90E-02	2.22E-01
chr14:75,278,923	FOS	T/C	T/T	T|C	9.20E-01	89|1	7.94E-01	398|2	6.82E + 00	542|3	288	2.68E-02	2.60E-01
chr4:119,217,669	USP53	A/G	A/A	A|G	1.08E-01	13	6.08E + 01	41|2	1.42E + 02	42|2	14	3.28E-02	8.99E-02
chr16:68,095,198	NFATC3	A/A	G/G	G|A	NA	NA	5.09E + 02	8|2	4.85E + 09	20|4	4	3.99E-02	1.39E + 00
chr4:48,586,725	FRYL	C/C	T/T	T|C	1.08E + 00	39|1	3.81E-01	67|1	4.48E + 00	127|2	53	4.10E-02	6.71E-01
chr11:62,524,481	AHNAK	T/A	A/A	A|T	2.45E + 01	14|1	2.34E + 00	111|1	2.14E-01	333|1	214	4.37E-02	2.89E-01
chr10:32,925,874	ITGB1	A/A	G/G	G|A	8.20E-02	178|2	3.23E + 01	484|3	2.70E + 02	912|6	482|1	9.04E-02	5.15E-01

^a^ ADGT (MOGT) denotes the genotype of adipocyte (macrophage) detected by SNP array

^b^ AlleleType (Count) denotes all alleles (the number of alleles) mapped at the corresponding location by the RNA-seq data on replicated samples

^c^ The Bayesian factor calculated at the specific locus for the corresponding sample

^d^ The fpkm value of the gene at ADMOexosome as quantified by Cufflinks

A search in PubMed for a role for our top 3 mRNA transfers, namely, *PIEZO1*, *RMRP* and *LAMC1* indicate their relevance in cellular communication processes.*PIEZO1* is an ion channel mediating mechanosensory transduction in mammalian cells [34]; *RMRP* is a lncRNA with multiple RNA targets [35] and *LAMC1* is a member of laminins(subunit gamma 1), which are a family of extracellular matrix glycoproteins [36]. Moreover, *LAMC1* is also a candidate gene for diabetic nephropathy [37]. Several other significant mRNA transfers include *HSPG2* and *SPTLC2*. *SPTLC2* encodes an enzyme involved in sphingolipid synthesis, which has been shown upon heterozygous deficiency to protect mice from insulin resistance [38]. Finally, the neuroblast differentiation-associated protein *AHNAK* is highlighted for its important role in the regulation of thermogenesis and lipolysis in WAT via b-adrenergic signalling. *AHNAK*−/− mice under a high-fat diet had enhanced insulin sensitivity and browning of the WAT depot [39]. Interestingly, *AHNAK* is a large plasma membrane protein of various functions including plasma membrane support, calcium signalling as well as regulated exocytosis via its key membership of a specialized vesicle, called enlargeosomes. Enlargeosomes are non-secretory, cytoplasmic vesicles competent of regulated exocytosis after rising intracellular calcium and contribute to plasma membrane repair and vesicle shedding [40, 41]. Finding that *AHNAK* mRNA is transferred from adipocyte to macrophages in exosomes suggests an additional level of complexity to the role of *AHNAK* in exocytosis.

Although it remains to be ascertained whether these mRNAs become functional proteins, evidence from others suggest that mRNAs transferred via exosomes do become functional proteins [4]. Thus these 25 genes (from both directions) are viable candidates for further studies with respect to communication between adipocytes and macrophages.

### Estimate the mRNA transfer rate between the two cell lines

To estimate the transfer rate and test the performance of our model at different transfer rates, we simulated mRNA transfers at various rates and performed analyses based on simulated data.

#### mRNA transfer simulation

**Fig. 3:**
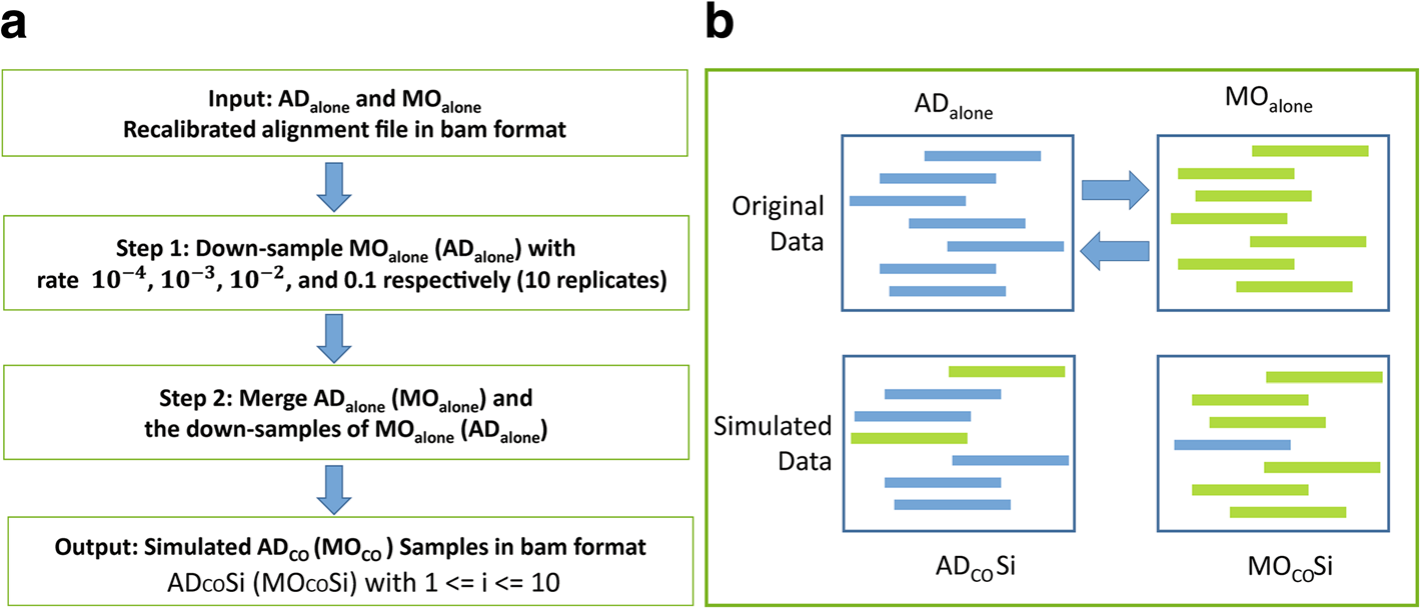
A pipeline to generate simulated data for adipocytes co-cultured with macrophages: (**a**) Steps used to generate simulated data. **b** A schematic to illustrate how we sampled reads from one type of cells and merged them with the reads from the other type of cells to simulate a sample with known mRNA transfers

The general work flow of the simulation process is shown in Fig. 3. Using simulated mRNA transfer from macrophages to adipocytes as an example, we randomly selected a predefined fraction of reads from MOalone and merged them with ADalone data. We then ran our previously described pipeline on the merged ADalone data to see how many loci with “transferred” reads could be correctly identified. For each predefined transfer rate, we performed 10 simulation runs (denoted as ${AD}_{co}^{Si}$ for 1 ≤ *i* ≤ 10with *S* indicates simulated data). Similarly, we constructed ${MO}_{co}^{Si}$ for 1 ≤ *i* ≤ 10 to study genetic transfer from adipocytes to macrophages. The simulation study has its unique advantage since we know exactly if a base mapped to a locus is from the donor cells or not, this would allow us to assess the performance of our pipeline of detecting mRNA transfer at different transfer rates.

#### Calling genetic exchange on simulated data and calculating accuracy

**Fig. 4:**
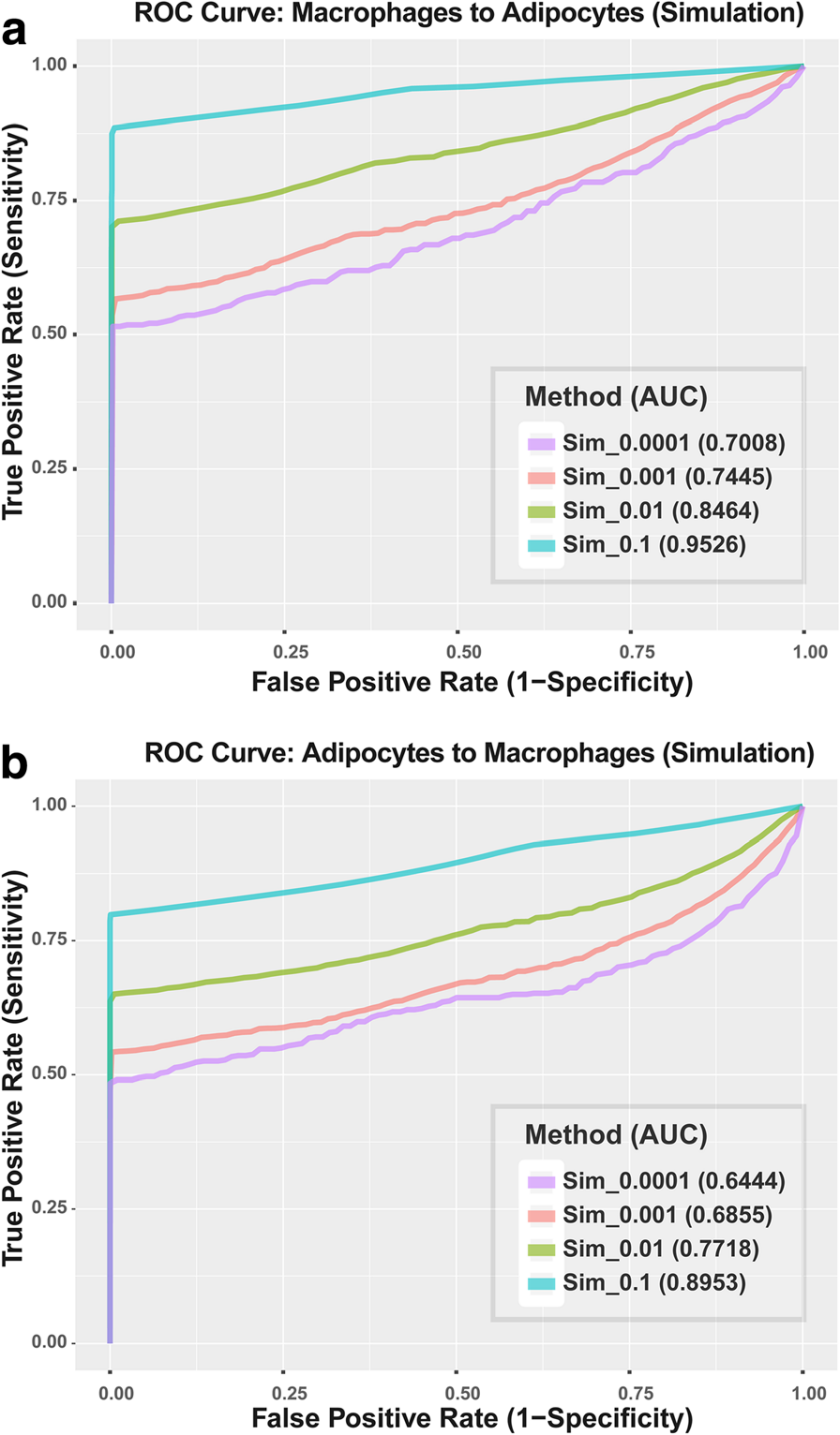
Performance of the Bayesian framework on simulated data with different transfer rates: (**a**) The ROC curves and AUCs for our method on simulated data from macrophages to adipocytes at 4 different transfer rates: 0.1, 0.01, 0.001, and 0.0001. **b** The ROC curves and AUCs for our method on simulated data from adipocytes to macrophages

We calculated the Bayesian factor at each “target” locus for the simulated samples. By merging the results from these samples and using Bayesian factor as a binary classifier, we created receiver operating characteristic (ROC) curves and calculated the area under curves (AUCs) of our method at 4 different transfer rates, i.e., 0.0001, 0.001, 0.01, and 0.1 (Fig. 4). As shown in Fig. 4, our method performs very well on identifying mRNA transfers at high transfer rates. At transfer rate 0.1, we achieved AUCs of 0.95 for transfer from macrophages to adipocytes and 0.90 for transfer in the opposite direction. The performance of our method decreases as transfer rate decreases. This is not surprising since when transfer rate decreases, the number of “alien” reads at each locus decreases, making signal to noise ratio to drop.

#### Estimation of mRNA transfer rate in the co-cultured samples

We provided a rough estimation of the transfer rate in our co-culture system by examining the difference in the overall distribution of Bayesian factor statistics obtained from the real co-culture data and the ones from simulated samples. The rationale underlying this analysis is that when the simulated transfer rate is similar to the real transfer rate, the difference between the two distributions should be minimal. It is of note that the numbers of reads are different in simulated samples and real co-cultured samples; this may cause the distribution of Bayesian factor statistics to change even when the underlying transfer rates are the same. To adjust for variation in the sample total read numbers to achieve a fair comparison, we down-sampled the reads data in ADco (MOco) such that it had roughly the same number of reads as the simulated co-cultured samples. We performed 10 runs of down-sampling, and generated 10 reads profiles denoted by ${AD}_{co}^{Ri}$ (${MO}_{co}^{Ri}$) (1 ≤ *i* ≤ 10 with *R* denotes real data), respectively for each transfer rate, i.e., 0.0001, 0.001, 0.01, and 0.1. We then calculated the Bayesian factor at each “target” locus for the down-sampled data. At a specific transfer rate, the overall distribution of Bayesian factor statistics in real co-culture is estimated by merging all the Bayesian factors from ${AD}_{co}^{Ri}$ (${MO}_{co}^{Ri}$) (1 ≤ *i* ≤ 10), and similar procedures were performed for simulated data. The Kolmogorov–Smirnov (KS) test was applied to compare the two distributions and we estimated the transfer rate to be the one with the lowest KS statistics. For both transfer directions, the minimal KS-statistics were achieved at transfer rate close to 0.001 (Table 4), indicating that the transfer rate in our co-culture system under quiescent condition was around 0.001.

**Table 4:** Estimate mRNA transfer rates in in vitro co-culture

Direction	Rates	Simulation			Real Co-culture			KS distance^c^
		# Loci^a^	#Sig Loci^b^	Sig Ratio	#Loci	#Sig Loci	Sig Ratio	
MO to AD	0.1	83,984	41,999	5.00E-1	80,969	972	1.20E-2	0.535
	0.01	73,838	6288	8.52E-2	74,056	841	1.14E-2	0.140
	0.001	73,037	513	7.02E-3	73,441	831	1.13E-2	0.047
	0.0001	72,958	107	1.47E-3	73,364	853	1.16E-2	0.066
AD to MO	0.1	225,698	61,305	2.72E-1	235,323	1293	5.49E-3	0.320
	0.01	215,598	7965	3.69E-2	223,395	1216	5.44E-3	0.058
	0.001	214,592	635	2.96E-3	216,059	1174	5.43E-3	0.025
	0.0001	214,509	58	2.70E-4	221,935	1202	5.42E-3	0.034

^a^ The number of overall loci checked in 10 simulated (down-sampled real) samples

^b^ The number of overall significant loci (Bayesian factor > = 20) in 10 simulated (down-sampled real) samples

^c^ The KS distance between the Bayesian factor distribution for simulated data and that for real co-culture data as calculated by ks.test in R

### Differentially expressed genes between cell lines cultured alone and co-cultured

To evaluate the impact of co-culturing to the transcriptome, we used cell lines cultured alone as control, and identified differentially expressed genes (DEGs) in the co-cultured system. We adopted two commonly used methods DESeq and EdgeR [23, 42] and summarized the results in Table 5. As can be seen in Table 5, the DEGs identified by the two methods were consistent even though DESeq inferred more differentially expressed genes than EdgeR. There were 575 and 142 DEGs (FDR ≤ 0.05) identified by both methods for adipocytes and macrophages respectively (Additional file 5: Dataset S4).

**Table 5:** Number of DEGs between cell lines cultured alone and co-cultured by DESeq and EdgeR (FDR ≤ 0.05)

	DESeq	EdgeR	Overlap	Fisher’s exact test *p*-value
AD_alone_ vs. AD_co_	1086	622	575	< 4.9E-324
MO_alone_ vs. MO_co_	168	171	142	2.1E-280

We performed function enrichment analysis of the DEGs using David tools (Version 6.7) [43] and the full results were provided in supplementary materials (Additional file 1: Table S2 for adipocytes and Table S3 for macrophages). The DEGs for adipocytes were mostly enriched in type 1 repeats in thrombospondin family, which are multimeric multidomain glycoproteins that function at cell surfaces and in the extracellular matrix milieu. *THBS1*, which encodes thrombosponin 1 was one of the genes found within this pathway significantly down-regulated in the co-cultured cells relative to the adipocytes cultured alone. *THBS1* is interesting with respect to type 2 diabetes as it has been shown to be elevated in the circulation in obese and insulin-resistance individuals [44] and loss of *THBS1* in mice, protects them from diet-induced weight gain and adipocyte hypertrophy [45]. Interestingly, the top 1, 3 and 6 genes identified in our transfer study, i.e., *ICAM1*, *PAPPA*, and *NPC1* are also significantly differentially expressed between ADalone and ADco with FDRs 1.02E-7, 1.36E-7, and 1.68E-5, respectively. *ICAM1* expression has been shown to relate with obesity and insulin resistance [46]. *NPC1* haploinsufficiency also promotes weight gain and metabolic features associated with insulin resistance [47].

In contrast to the adipose tissue, the DEGs identified by comparing MOco vs. MOalone are mostly enriched for transmembrane proteins, immune response pathways such as leukocyte activation, and EGF-like domains. Most occurrences of the EGF-like domain are found in the extracellular domain of membrane-bound proteins or in proteins known to be secreted. The EGF receptor family is in part involved in Notch signaling, which controls cell-cell communication [48]. It is of note that a lot of studies have shown that ECM modulates epidermal growth factor receptor activation and leukocytes [49, 50]. Thus, the differential expressed genes could be some down-stream effects of the transferred transcripts from adipocytes to macrophages.

## Discussion

There is accumulating evidence that exosomes via horizontal transfer (i.e., the movement of genetic material between cells) of genetic information can play a key role in cell-to-cell communications [4–6, 51, 52]. Numerous studies have thus focused on providing a comprehensive characterization of the content of EVs and these efforts have led to the creation of databases, such as EVpedia and Vesiclepedia [53, 54], which record molecules (proteins, mRNAs, microRNAs or lipids) observed within these vesicles. However, being identified in exosomes is not necessarily indicating that the RNAs or proteins will be transferred into other cells. Thus in the present study, we complement these efforts by providing a computational approach to identify genetic material that has been transferred between two in vitro co-cultured cells mediated by EVs. Next generation sequencing of cellular genetic material which differed between the co-cultured cell types was used as the finger print to place donor-derived mRNAs at the scene of the recipient’s cellular RNA pool. In comparison to other labelling technologies, using DNA sequence polymorphism as marker has its advantages since they are naturally occurring and introduce no artificial modifications to the biological systems. On the other hand, we are limited to loci with polymorphisms for mRNA transfer detection, and therefore this approach while at genome-scale provides semi-whole genome coverage.

In comparison of our identified 25 genetic transfers (in both directions) to those catalogued in the exosome database, ExoCarta (~1700 distinct human mRNAs across various tissues sources) [55], we identified 7 mRNAs in common (*ITGB1, NFATC3, SOD2, FOS, AHNAK, LAMC1,* and *SPTLC2*), all of which are in the direction from adipocytes to macrophages (with *p*-value 2.45E-3). The overlap is significant despite the diversity seen in exosome cargo depending on the cell type under study as well as the cellular state. Nonetheless this serves to further underlie the importance of characterization of EV contents. While our method has general applicability, as described below, we perceptively chose to apply this to co-cultures of macrophage and adipocyte given the precedence in the literature that there is crosstalk between adipocytes and immune cells in the adipose tissue that contributes to metabolic dysregulation and obesity [56]. The function of the mRNAs transferred range from protein coding to transcriptional regulators and thus the impact on the recipient cell could vary greatly if they are translated into functional units. Although in our study we did not assess this, evidence from other studies suggest the transferred mRNA can be functional in the recipient cell. From this perspective, it is of great interest to see that one of the mRNAs we identify as transferred from adipocyte to macrophage, as well as confirmed in ExoCarta, is AHNAK. Given the most recent identification that AHNAK in the regulation of thermogenesis and lipolysis in WAT via β-adrenergic signalling makes this observation of AHNAK mRNA transfer in adipocyte exosomes to macrophages very relevant to the new field of extracellular vesicle biology and the possible impact for new therapeutics for T2D. It also highlights the value of this computational approach in generating novel hypothesis.

Currently our method is optimized for detecting nucleic acid exchanges between two cells lines at loci with known polymorphisms. It is known that exosome RNA contains not only mRNA, but also non-coding RNA species such as small microRNAs. For example, by analysing miRNA expression levels in a variety of cell lines and their derived exosomes, Guduric-Fuchs et al. found that miRNAs like miR-150, miR-142-3p, and miR-451 preferentially enter exosomes [57]. Huang et al. characterized the human plasma-derived exosomal RNAs by deep sequencing [58]. Although we did sequence miRNAs from the cells alongside total mRNA, we could however, not find sufficient genetic diversity amongst the miRNAs between the adipocyte and macrophages in order to allow for the identification of transfers via genetic differences mainly due to short length and relatively small number of miRNA species that could be detected.

Although accumulating evidence supports the important role of exosomes/EVs for mediating the cell-to-cell communication and exchange of genetic information, exosomes or lipid vesicles in general may not be the only mechanism for RNAs to transfer between cells. For example, it has been demonstrated that miRNA can be protected in the extracellular environment by forming complex with high-density lipoproteins (HDL) and RNA-binding proteins [59, 60]*.* Since our experimental design did not exclude other mechanisms of genetic material transfer besides EVs, we consider the identified transfers are likely mediated by exosomes but could also be contributed by other mechanisms. There are a few possibilities that can lead to false discoveries. First, reads from RNA-seq contain errors due to the technical limitation of the next generation sequencing technology [61]. For example, the raw quality scores of Illumina sequencing are calculated from the signal intensity, which do not always accurately represent the true error rate. Our study is particularly sensitive to this error rate since the transfer rate of transcripts is also quite low in this study as indicated by our simulation study. When the transfer rate is low (as seen in our experiment), the low signal/noise ratio could be the major factor for the high FDRs. Second, all the aligners may have some mapping errors. A comparison study with various sequence aligners shows that STAR-2pass with annotation has the best alignment performance but is still not completely error free [62]. Third, there are also genotyping error by SNP-arrays [63]. Finally, there are a few biological mechanisms such as RNA editing which will cause false discovery in our method.

Our simulation study has shown that our method has its ideal performance when the transfer rates between the two in vitro co-cultured cell lines are high. Inducing exosome secretion could be done via chemical means, such as altering cellular ceramide levels [64]. Our methods also require a good level of genetic diversity considering the donor and recipient systems. Optimization of genetic diversity can be done using cultures of cells that are knowingly from different genetic individuals such as in our experimental design. Other experimentally relevant systems to optimize genetic diversity, include investigations surrounding human derived exosomes (e.g., from plasma) and their function by injecting into the mouse [65]. In this case, one could survey the mouse tissues in order to identify tissues which have been impacted by the nucleic acid cargo of the donor exosome. Another naturally expressing genetic diversity is seen in cancers. It has been known that cancer cells secrete exosomes including during cell migration [66] and invasion [67]. Importantly, tumour derived microvesicles have been identified to contain mutated and amplified oncogenic DNA sequences and potentially have a role in genetic communication between cells as well as provide a potential source of tumour biomarkers. Thus our approach, we predict, would be highly amenable to identifying the genetic materials transferred between cancer and surrounding or distant normal cells [52] and further highlights the overall potential impact of this methodology.

## Conclusions

In this study we present a novel systematic framework to call genes involved in the process of mRNA exchange between two co-cultured cell lines of different genetic backgrounds and investigate the role of exosomes as a vehicle in mediating the exchange. The systematic framework includes a protocol to perform quality control, alignment, mapping, and base call recalibration on the raw SNP array and RNA sequencing reads data, a Bayesian model to evaluate the significance of genotypic variation of a cell line under in vitro co-culture, and a method to estimate the rate of false discovered loci involving in the transfer process. By applying the framework to a co-culture between adipocyte and macrophage cell lines, we identified with high confidence, 8 mRNAs being transferred from macrophages to adipocytes and 21 mRNAs transferred in the opposite direction. These mRNAs represent biological functions including extracellular matrix, cell adhesion, glycoprotein, and signal peptides. We also estimate the transfer rate to be 0.001 in both directions. Our work provides a novel solution to studying EV mediated mRNA transfers between cells and this work is able to be extended to in vivo studies as well.

## Additional files

Additional file 1: A next generation sequencing based approach to identify extracellular vesicle mediated mRNA transfers between cells. (DOCX 1282 kb)

Additional file 2: Dataset S1. Informaiton of filted loci from adipocyte to macrophage and from macrophage to adipocyte. (XLS 3896 kb)

Additional file 3: Dataset S2. Details of mRNA transfer analysis from macrophage to adipocyte. (XLSX 1903 kb)

Additional file 4: Dataset S3. Details of mRNA transfer analysis from adipocyte to macrophage. (XLSX 6308 kb)

Additional file 5: Dataset S4. Differential genes between adipocyte cultured alone and co-cultured with macrophage and between macrophage alone and co-cultured with adipocyte. (XLSX 96 kb)

## Abbreviations

AD: Adipocyte
AUCs: Area under curves
CDCs: Cardio sphere-derived cells
DEGs: Differentially expressed genes
EVs: Extracellular vesicles
exRNA: Extracellular RNA
FDR: False discovery rate
FPKM: Fragments per kilobase of transcript per million
GO: Gene ontology
HDL: High-density lipoproteins
IGFBPs: Insulin-like growth factor binding proteins
IGV: Integrative genomics viewer
KO: Knockout
KS: Kolmogorov–Smirnov
MO: Macrophage
MVs: Microvesicles
NGS: Next generation of sequencing
PAPP-A: Pregnancy-associated plasma protein-A
PCA: Principal component analysis
QC: Quality control
RNA-seq: RNA sequencing
rRNAs: Ribosomal RNAs
SNP: Single nucleotide polymorphism
T2D: Type 2 diabetes
