# Supplementary material for: A next generation sequencing based approach to identify extracellular vesicle mediated mRNA transfers between cells
Source: BMC Genomics. 2017 Dec 22;18:987. doi: 10.1186/s12864-017-4359-1 (PMC5741891; doi:10.1186/s12864-017-4359-1)
Supplement: Supplementary file 1 — A next generation sequencing based approach to identify extracellular vesicle mediated mRNA transfers between cells. (DOCX 1282 kb) [file 12864_2017_4359_MOESM1_ESM.docx]

Supplementary Material

**A next generation sequencing based approach to identify extracellular vesicle mediated mRNA transfers between cells**

Jialiang Yang^1,2^, Jacob Hagen^1,2^, Kalyani V. Guntur^3^, Kimaada Allette^1,2^, Sarah Schuyler^1,2^, Jyoti Ranjan^3^, Francesca Petralia^1,2^, Stephane Gesta^3^, Robert Sebra^1,2^, Milind Mahajan^1,2^, Bin Zhang^1,2^, Jun Zhu^1,2^, Sander Houten^1,2^, Andrew Kasarskis^1,2^, Vivek K. Vishnudas^3^, Viatcheslav R. Akmaev^3^, Rangaprasad Sarangarajan^3^, Niven R. Narain^3^, Eric E. Schadt^1,2^, Carmen A. Argmann^1,2,*^, Zhidong Tu^1,2,*^

^1^ Institute of Genomics and Multiscale Biology, Icahn School of Medicine at Mount Sinai, NY, 10029, USA

^2^ Department of Genetics and Genomic Sciences, Icahn School of Medicine at Mount Sinai, NY, 10029, USA

^3^ BERG, LLC, Framingham, MA, 01701, USA

* To whom correspondence should be addressed. Email: [zhidong.tu@mssm.edu](mailto:zhidong.tu@mssm.edu), and carmen.argmann@mssm.edu.

**Supplementary Methods**

### Cell culture

*Human primary preadipocyte culture and differentiation.* Cryopreserved human primary omental Pre-adipocytes (OM062106A) were plated at 700,000 cells/well of a 6-well cell culture plates, in Promocell Preadipocyte Basal Media (Cat# C-27410) supplemented with Promocell growth supplements without antibiotics. The following day, preadipocytes were differentiated into adipocyte using Promocell Preadipocyte Basal Media with 25mM glucose and supplemented with 10% FBS, Insulin (0.5 µg/ml), d-Biotin (8 µg/ml), Dexamethasone (400 ng/ml), IBMX (44 µg/ml), L-Thyroxine (9 ng/ml), and Ciglitazone (3 µg/ml) for 3 days. On day 3, media was changed and replaced with Promocell Adipocyte Nutritional Media consisting of Adipocyte Basal media (Cat# C-27431) supplemented with 10% FBS, Insulin, Dexamethasone and Biotin. Adipocyte Nutritional media was changed on day 5, day 7, day 9 and day 11 of adipocyte differentiation.

*U937 monocytes culture and differentiation in macrophages.* U937 monocytes were plated and differentiated into macrophages on polyester inserts of the Corning 6-well plates at 350,000 cells/well using RPMI-1640 media containing 20nM of PMA supplemented with 10% FBS and antibiotics. After 3 days of differentiation, U937 macrophages were rested in RPMI-1640 media containing 10% of FBS and antibiotics for 48 hours.

*Adipocyte.* For adipocytes cultures, cells were washed once with PBS. Cells were then trypsinized (1ml of trypsin at 37ºC for 5 minutes) and resuspended in Adipocyte Basal Media with high glucose (25mM) supplemented with 10% serum. Cells were pelleted at 1000rpm for 5 minutes at 22ºC. Cell pellet was then resuspended with PBS and pelleted again at 1000 rpm for 5 minutes at 22ºC. PBS was then aspirated off, cells were frozen in liquid nitrogen and stored at -80ºC.

*Adipocyte macrophage co-culture.*

*The transwell system*: Transwell membrane system is a cell and tissue culturing technique that produces an environment that resembles the in vivo state as closely as possible to enable the growth of certain cell types. It uses permeable supports with microporous membranes for culturing these cells. These permeable supports are very useful in culturing polarized cells since they permit cells to uptake and secrete molecules on both basal and apical surfaces and thereby carry out transport, absorption, secretion, and metabolic activities in a more natural fashion. Cellular differentiation can also proceed to higher levels resulting in cells that morphologically and functionally better represent their in vivo counterparts. Unique self-centred hanging design of these transwell membranes prevents medium wicking between the insert and outer well. It permits access to the lower compartment through windows in the insert membrane. The suspended design allows for undamaged co-culturing of cells in the lower compartment. The polyester membranes used for these experiments have the best overall chemical resistance. These membranes are compatible with many alcohols, amines, esters, ethers, ketones, oils and some solvents including many halogenated hydrocarbons and DMSO.

*Specifications of the transwell system used:*

Microscopically transparent polyester membrane is TC-treated for optimal cell attachment and growth. These transwell multi-well plates with polyester membrane inserts provide better visibility under phase contrast microscopy and allow assessment of cell viability and monolayer formation. The transwell multi-well plates were manufactured by Corning™ 3450 with **Catalog No.**07-200-170, which have pore size of 0.4um. This is the same system as used by Garcia et al.[[1](#_ENREF_1)].

*Adipocyte macrophage co-culture Experiments:*

On day 12 of adipocyte differentiation, adipocytes and macrophages were washed once with serum free Adipocyte Basal Media (Promocell Cat# C-27431) with high glucose (25mM). For co-culture, U937 containing polyester inserts were placed on top of adipocyte containing 6-well plates. Cells were co-cultured in Promocell Adipocyte Basal Media with high glucose (25mM) without serum and supplements for 48 hours. A total of 1.5 ml of media was added to the insert (macrophage) and 2.5 ml of media was added to the well (adipocyte). Each experimental replicate consisted of six separate wells, the contents of which were pooled at the end of the experiment. The adipocyte-macrophage co-culture was independently replicated three time (n=3).

Following 48 hours of co-culture, media from the insert and from the wells were collected. Samples were briefly spun down for 5 minutes at 5,000 rpm, frozen in liquid nitrogen and saved at -80ºC. For macrophages, PBS was added to the inserts containing macrophages and was incubated at 37ºC for 5 minutes allowing macrophages to lift-up from the plate. Cells were then collected and pelleted at 1000 rpm for 5 minutes at 22ºC. Cell pellet was then resuspended with PBS and pelleted again at 1000 rpm for 5 minutes at 22ºC. PBS was then aspirated, cells were frozen in liquid nitrogen and stored at -80ºC.

### Biospecimen Collection and Processing

*Exosome isolation.* Exosomes were isolated using sequential centrifugation essentially as described by Thery et al. [[2](#_ENREF_2)]. All spin steps were at 4 ^o^C. Briefly, samples were spun at 10 000 x g for 30min. Replicate samples were pooled into 15ml canonical tubes, and then concentrated to approximately 750ul using Amicon (Sigma Aldrich) ultra-centrifugal filter units (100kDa). The concentrated media was then spun at 100 000 x g for 120 mins. The supernatant was removed, and the pellet was resuspended in RNAse free water. The resuspended pellet was then spun for another 120 min at 100 000 x g. The supernatant was removed and the pellet was resuspended in 50ul RNAse free water. Half the volume was used for western blotting and the other half was used for RNA extraction. Western blotting was done following the protocol as previously described [[3](#_ENREF_3)], except that no reducing agent or homogenization step in RIPA buffer was done. Primary antibodies were human CD81 and CD9 (System Biosciences, ExoAB Antibody kit). Western blot analysis of purified exosomes are shown in Fig. S7.

*RNA isolation from cell pellets and exosomes.* Total RNA was isolated from cell pellets under four conditions (i.e., adipocytes cultured alone, macrophages cultured alone, adipocytes co-cultured with macrophages, and macrophages co-cultured with adipocytes) and from exosomes in the media under three conditions (i.e., adipocytes cultured alone, macrophages cultured alone, and co-culture) and were sequenced by Illumina HiSeq sequencing. Total RNA from cell pellets and exosomes was extracted using the miRNeasy kit (Qiagen) and RNA was eluted into RNAse free water. Total RNA from exosomes given the low yield was subject to a separate amplification protocol as described in the next section.

*Clontech Smart-Seq v4 Ultra Low Input Exosome RNA Sample Preparation.* Exosomal RNA samples were subject to first strand cDNA synthesis using the Clontech Smart-Seq v4 Ultra Low Input kit procedure. Briefly, 3’ SMART-Seq CDS Primer II A was added to each exosome RNA sample and incubated at 72 °C for 3 minutes. A master mix was prepared by mixing; per reaction, 4μl 5X Ultra Low First Strand buffer, 1μl SMART-Seq v4 Oligonucleotide (48uM), 0.5ul RNase Inhibitor (40U/ul), and 2μl per reaction of SMARTScribe Reverse Transcriptase. Master mix was added to each sample and were incubated for 3 minutes at 72 °C, then placed on ice for 2 minutes. The tubes were placed on thermal cycler 42 °C and ran according to the following program; 42 °C for 90mins, 70 °C for 10mins. A cDNA amplification step was then applied to 20μl of first strand cDNA product and ran according to the following program, 95 °C for 1min, X cycles (refer to Table S4) (98 °C for10sec, 65 °C for 30sec, 68 °C for 3 mins), and 72 °C for 10mins. In the case of exosome samples, the total RNA input table describes the correlation between number of PCR cycles and input mass of RNA that we used in the work presented in this manuscript.

*Purification of Amplified cDNA using Agencourt Ampure Beads.* The amplified cDNA exosomal RNA product was then subjected to a purification step using Agencourt Ampure Beads. Briefly, 50μl of Ampure beads were added to each double stranded product tube and incubated for 10mins. The tubes were placed on magnetic separation device for 5 mins or until solution was clear. The supernatant was removed and discarded. With the tubes on the magnetic separation device, the beads were washed twice with 200ul 80% ethanol and the supernatant was carefully removed and discarded. The tubes were placed on the magnetic separation device, the remaining ethanol was removed and air-dried for 5 minutes. 17μl of elution buffer was added, mixed thoroughly to resuspend the beads and incubated at room temperature for 2 minutes. The tubes were placed on the magnetic separation device for 1 minute or until the solution was completely clear. The supernatant was removed and place in a clean tube. Each double stranded cDNA product was assessed for concentration and fragmentation quality. The concentration for each sample was obtained using Qubit fluorometry and the product fragmentation was validated using the Agilent 200 Bionalyzer (see Fig. S8) prior to further molecular methods for downstream experiments.

### Genotyping and Quality Control

*Genotyping.* DNA was extracted from pellets collected from cells cultured alone using QiaAmp DNA blood mini kit (Qiagen). DNA from each condition was genotyped by the Illumina HumanOmni2.5Exome-8 BeadChip. To avoid randomness, two replicates were genotyped for each cell line, namely Adipocyte B1, Adipocyte B2, Macrophage B1, and Macrophage B2.

*Quality control on genotyping data.* We first performed quality control (QC) to filter out low quality genotype data. Specifically, we filtered out the loci whose alleles contain characters other than A, G, C, or T, or whose chromosome and position are marked as 0. We then compared the similarity or difference between Adipocyte B1 and B2, between Macrophage B1 and B2, and between adipocyte and macrophage (Main text Fig. 2). To be consisted with the RNA-seq reads which are mapped according to reference genome Hg38, we then mapped the genotype at each locus such that it will be matched to Hg19 positive strand (using the support file “HumanOmni2-5Exome-8-v1-1-strand-report-fdtp-a.txt” downloaded from the Illumina website <http://support.illumina.com/downloads/humanomni2-5exome-v1-1-product-support-files.html>) and then lifted over the coordinates to Hg38 using the UCSC tool (<https://genome.ucsc.edu/cgi-bin/hgLiftOver>).

### RNA Sequencing

*Library preparation and sequencing.* cDNA library was prepared cellular RNA using a non-strand specific protocol with ribozero depletion of rRNA. Sequencing was paired-end at a length of 100nt. For each sample, we obtained higher coverage than regular RNA-seq. We obtained particularly higher coverage for the co-culture samples to allow additional power to identify variants from sequencing data. Co-cultured samples were sequenced at a depth of about 75 million – 90 million while the alone samples were sequence at about 37 million – 45 million.

*Quality control on RNA-seq data.* We filtered ribosomal RNAs (rRNAs) using SortMeRNA [[4](#_ENREF_4)] and trimmed Illumina adaptors using Trimmomatic [[5](#_ENREF_5)]. We then used FastQC to do a quality check on filtered data for each sample. This software checks for criteria including base quality, GC content, sequence length distribution, sequence duplication levels, and so on.

### Alignment of pair-ended RNA reads

The short reads were mapped to the reference genome (Hg38) by STAR 2-pass with annotation GENCODE V21 and only the uniquely mapped reads were kept. The resulted SAM files were put through the usual Picard processing steps: fixing mate, adding read group information, sorting, marking duplicates, and indexing. GATK tool SplitNCigarReads was used to split the reads into exon segments and hard-clip any sequences overhanging into the intronic regions, and GATK tools ReassignOneMappingQuality was used to reassign mapping qualities [[6](#_ENREF_6)]. In addition, a base call recalibration was done by using GATK tool BaseRecalibrator against SNP data downloaded from 1000 genome project (version All_20160527). Samtools mpileup was used to retrieve the base profile mapped at each locus [[7](#_ENREF_7)].

### Gene expression

We used the Tophat/Cufflinks pipeline [[8](#_ENREF_8)] to call Fragments per Kilobase of Exon per Million Fragments Mapped (FPKM) of each gene against Hg38 reference genome. We used the annotation file GENCODE V21 throughout this study. In addition, we first filtered genes that have more than 80% zero expression among samples and then performed inverse-quantile normalization for genes in each sample. A principal component analysis was then employed to check the sample distribution based on normalized gene expressions (Fig. S2).

### SNV call on cell pellets

We applied the GATK pipeline to call single nucleotide variants (SNV) on all samples. The SNPs being called with read depth greater than or equal to 10 and passing the filtering criteria, i.e., (1) filtering clusters of at least 3 SNPs that are within a window of 35 bases and (2) filtering based on Fisher Strand values (FS > 30.0), and (3) filtering the “LowQual” variant with quality score less than 30 for each sample were used to construct the heat map Fig. S3.

### Down Sampling

The down sampling was down by applying “Samtools –s”, in which each reads is flipped with the given probability to join the down-sample [[7](#_ENREF_7)][.](file:///C:\Users\Jialiang\Desktop\2-28-2017Submission\Science-Advances-Manuscript-Template.doc#_ENREF_15)

**Supplementary Figures**

**Fig. S1.** A cartoon illustrates the experimental design on cell co-culture.


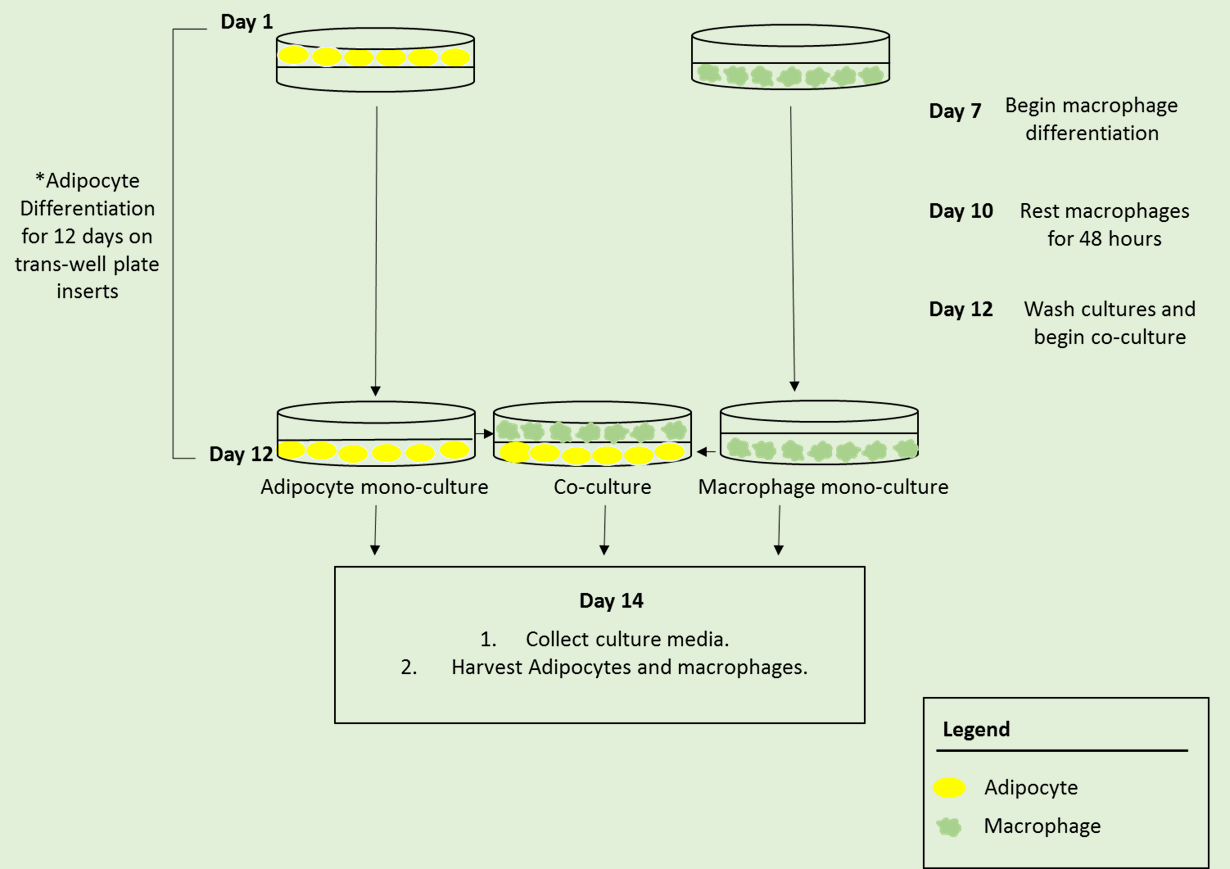


**Fig. S2.** Principal Component analysis on gene expression of 10 cell line samples and 2 exosomes


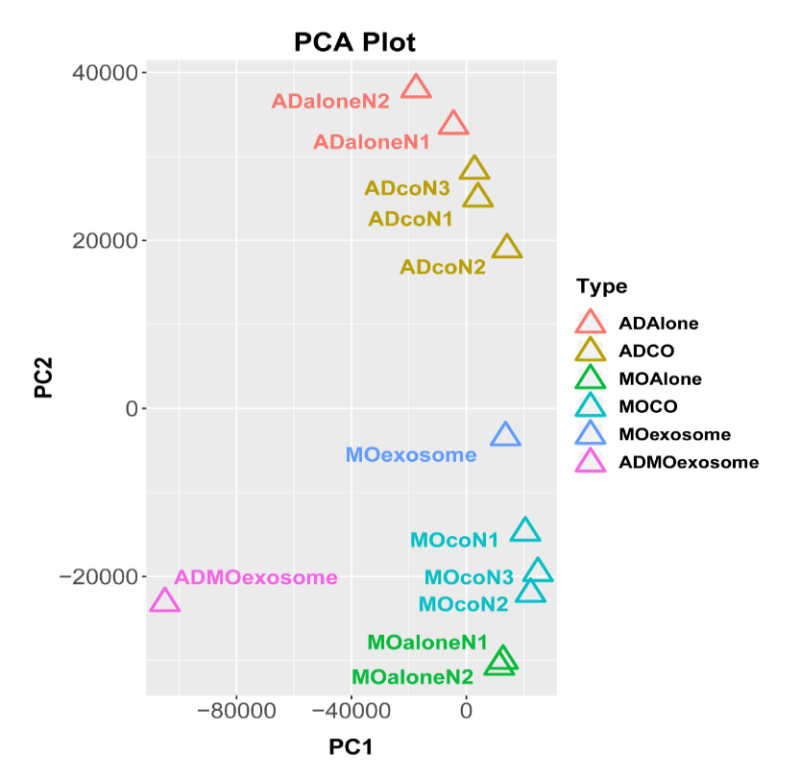


**Fig. S3.** Consistency of SNP calling among 10 cell line samples and 2 exosomes.


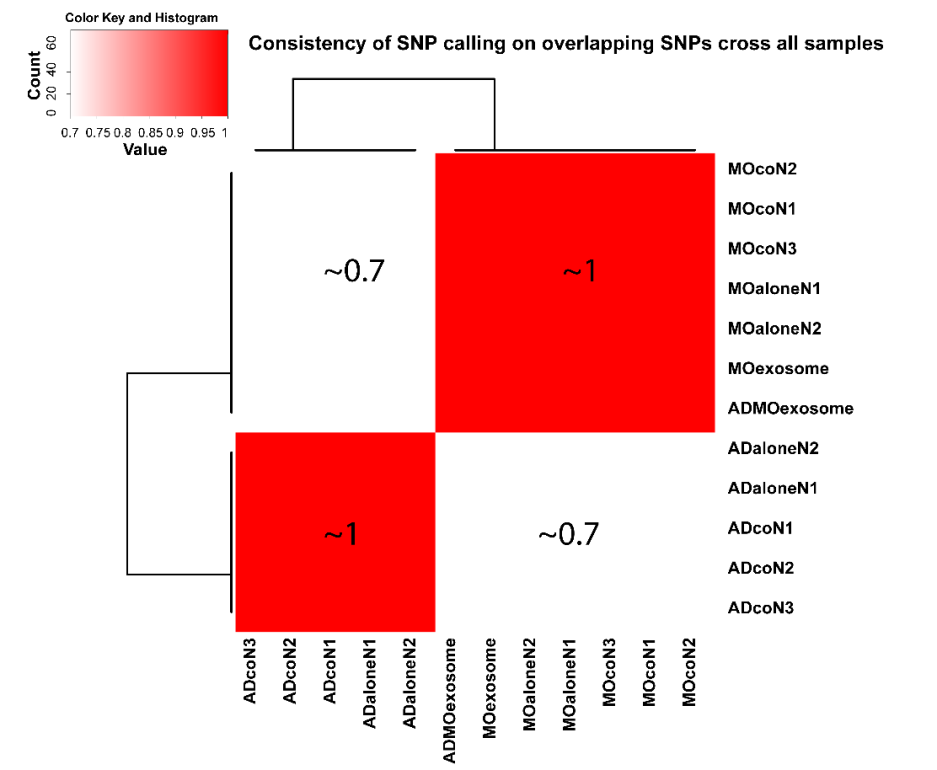


**Fig. S4.** A figure to illustrate the notation used by the Bayesian model


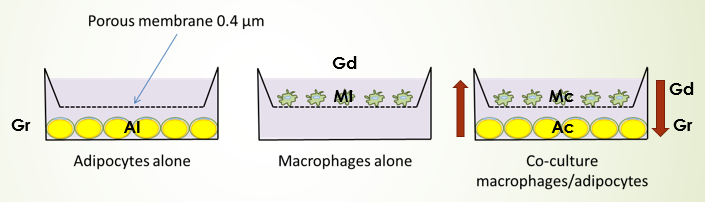


We denote the read data at a particular genome position from these four profiles by$A_{I}$, $A_{C}$, $M_{I}$, and $M_{C}$, corresponding to adipocyte alone, adipocyte co-cultured, macrophage alone, and macrophage co-cultured cells, respectively, and $G_{d}$ and $G_{r}$ be the genotypes of donor cells and receptor cell respectively.

**Fig. S5.** IGV plot of the alignment of MO_alone_N1-N2, AD_co_N1-N3, and AD_alone_N-N2 at chr19:10286547 (*ICAM1*)

**
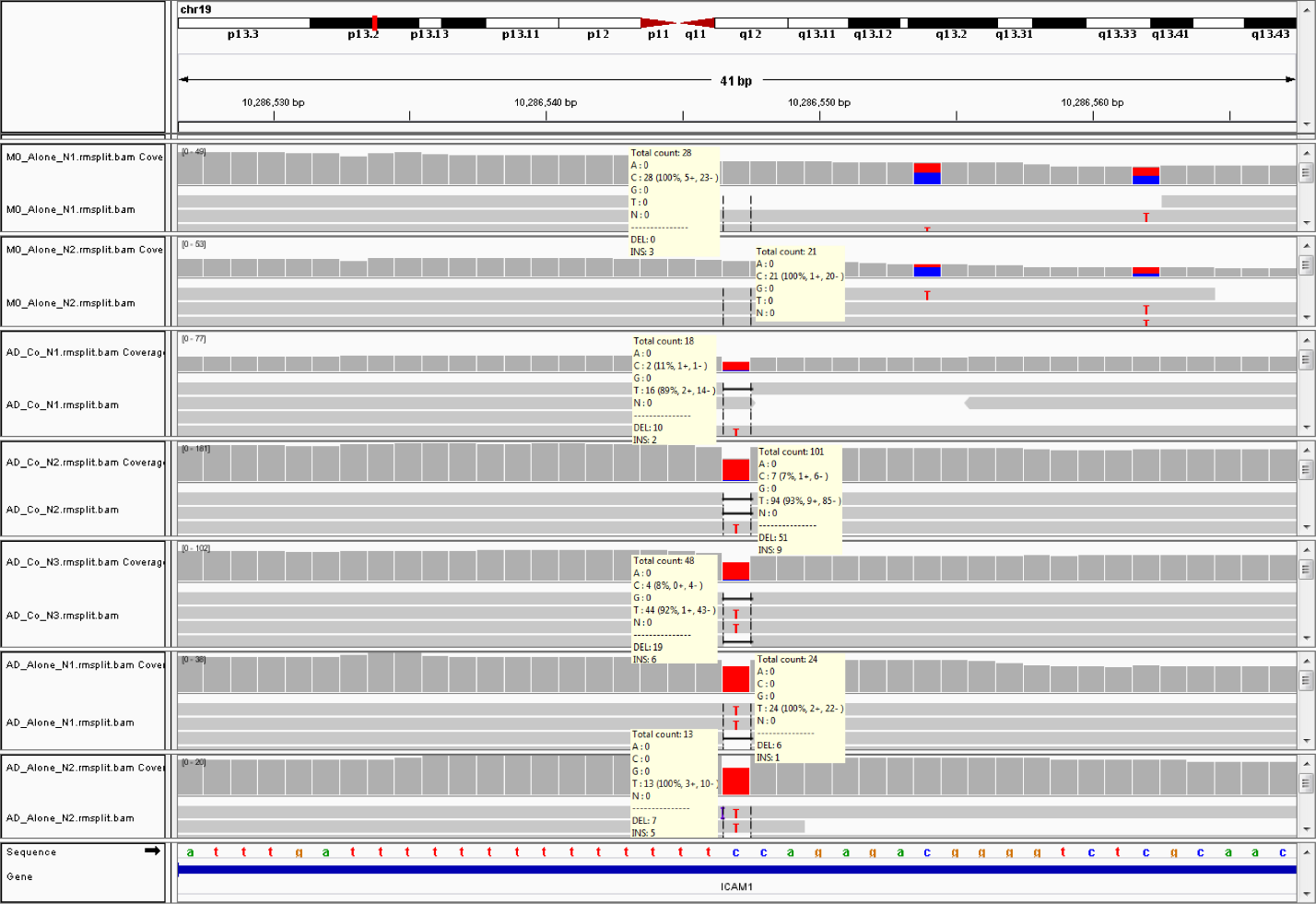
**

**Fig. S6.** IGV plot of the alignment of AD_alone_N1-N2, MO_co_N1-N3, and MO_alone_N-N2 at chr6:75666836 (*SENP6*)

**
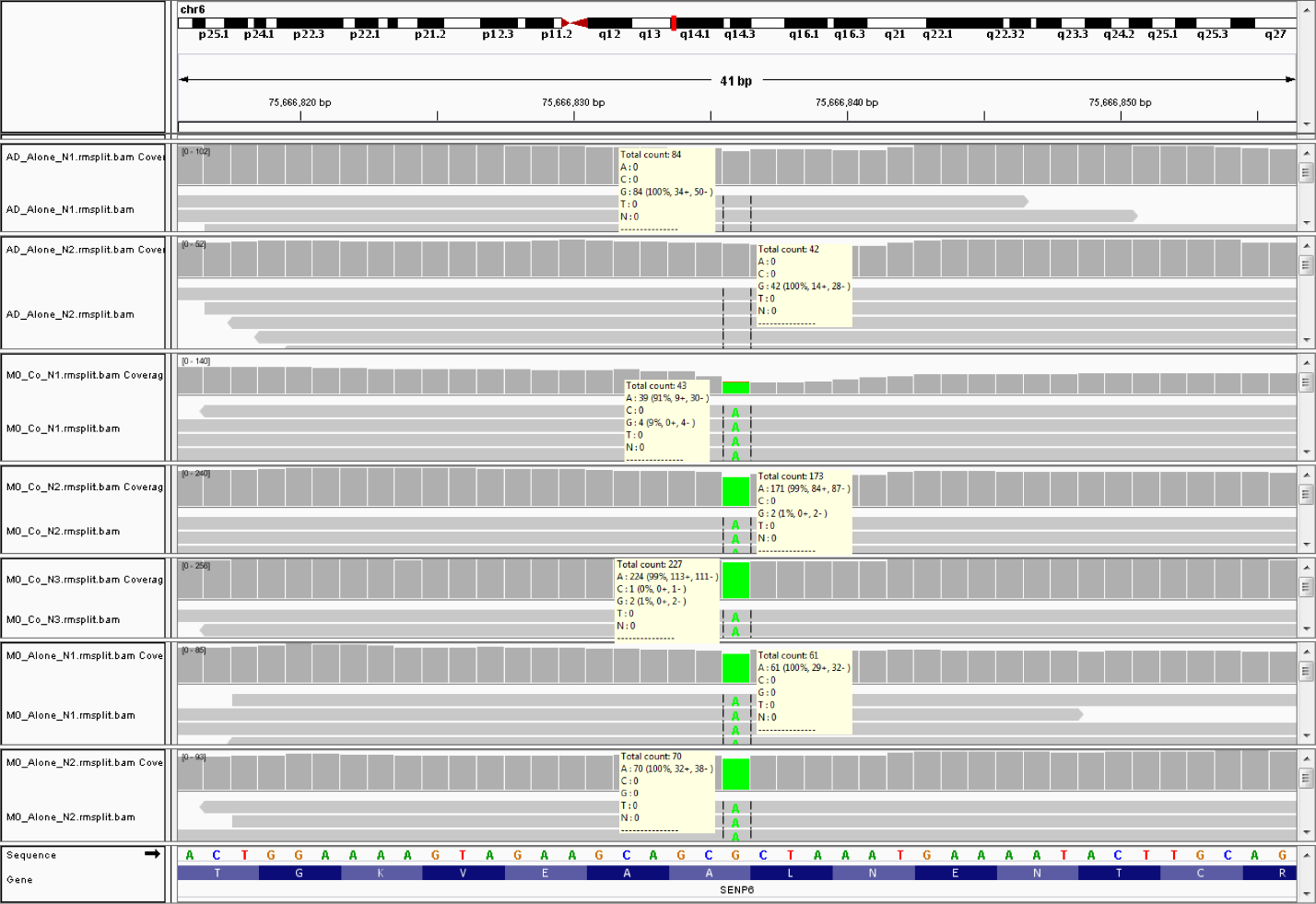
**

**Fig. S7.** Analysis of known markers of exosomes by Western Blot analysis


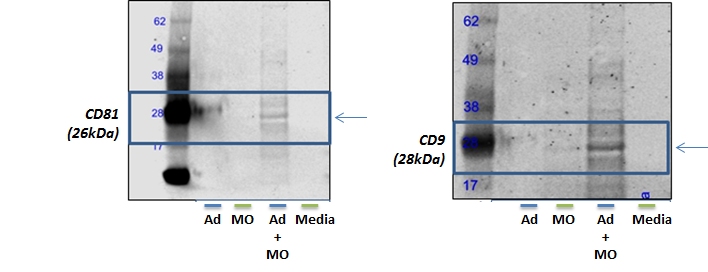


**Fig. S8.** Bioanalyzer analysis of amplified cDNA exosome RNA product


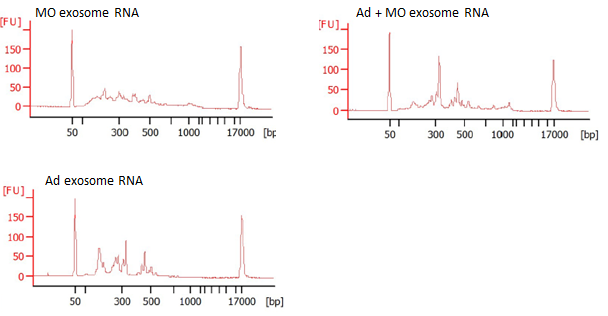


**Supplementary Tables**

**Table S1.** Summary of alignments information of RNA-seq on 10 cell samples and 3 exosome samples

|  | **# Input pairs** | **# Uniquely mapped pairs** | **Mapping rate** |
| --- | --- | --- | --- |
| AD_alone_N1 | 24167459 | 22917669 | 94.83% |
| AD_alone_N2 | 13609924 | 12942821 | 95.10% |
| AD_co_N1 | 26774747 | 24222315 | 90.47% |
| AD_co_N2 | 50448911 | 47244012 | 93.65% |
| AD_co_N3 | 28028914 | 25825185 | 92.14% |
| MO_alone_N1 | 26884190 | 24706464 | 91.90% |
| MO_alone_N2 | 27352457 | 25520323 | 93.30% |
| MO_co_N1 | 31295700 | 28224254 | 90.19% |
| MO_co_N2 | 56920942 | 53294626 | 93.63% |
| MO_co_N3 | 89974998 | 83520570 | 92.83% |
| AD_exosome_ | 528215 | 45196 | 8.56% |
| MO_exosome_ | 56774245 | 48640889 | 85.67% |
| ADMO_exosome_ | 11468406 | 9270314 | 80.83% |

**Table S2.** Function enrichment analysis on differential genes between AD_alone_ and AD_co_

| **Category** | **Term** | **Count** | **Genes** | **Benjamini** |
| --- | --- | --- | --- | --- |
| SMART | SM00209:TSP1 | 9 | WISP2, WISP1, C9, UNC5B, THSD4, ADAMTS1, THBS1, CYR61, ISM1 | 3.33E-02 |
| SP_PIR_KEYWORDS | oxidoreductase | 28 | LDHC, HSD17B10, ME2, CYP1B1, ADH1B, ALDH1L2, GPD1L, AKR1C2, MTHFD2, TP53I3, TYR, CYP27B1, HMOX1, CYP26B1, HSD17B6, PIWIL2, IDH1, AKR1C1, NOX4, BCKDHB, FOXRED2, CYP20A1, CYP4V2, DHFR, CYP27A1, ALDH1B1, PHGDH, AOC2 | 5.40E-02 |
| SP_PIR_KEYWORDS | cell adhesion | 22 | ICAM1, FLRT2, CLSTN2, NRXN2, ICAM5, ITGA10, PCDH9, ITGB3, PCDHAC2, WISP2, OMD, WISP1, COL14A1, CD36, CD34, CNTN2, THBS1, PDZD2, MFAP4, ADAM12, CYR61, CDH23 | 1.06E-01 |

**Table S3.** Function enrichment analysis on differential genes between MO_alone_ and MO_co_

| **Category** | **Term** | **Count** | **Genes** | **Benjamini** |
| --- | --- | --- | --- | --- |
| SP_PIR_KEYWORDS | transmembrane  protein | 18 | C5AR1, HLA-DRB1, SELL, FLT3, TRGC2, FURIN, ITGAM, CD74, CD163, CD38, PLSCR1, ADRB2, FCAR, CXCR4, P2RY2, KCNE1, HBEGF, HLA-DRA | 1.65E-04 |
| GOTERM_BP_FAT | GO:0046649~lymphocyte activation | 10 | KLF6, BLM, CXCR4, LAX1, FLT3, RELB, BCL3, SLAMF1, CD74, ITGAM | 6.61E-03 |
| GOTERM_BP_FAT | GO:0042325~regulation of phosphorylation | 15 | C5AR1, BLM, SOCS3, CD74, IL31RA, TRIB1, SPRY1, ADRB2, CERKL, CXCR4, DGKD, LAX1, JUN, IL1B, PRKACB | 7.05E-03 |
| GOTERM_BP_FAT | GO:0019220~regulation of phosphate metabolic process | 15 | C5AR1, BLM, SOCS3, CD74, IL31RA, TRIB1, SPRY1, ADRB2, CERKL, CXCR4, DGKD, LAX1, JUN, IL1B, PRKACB | 7.36E-03 |
| GOTERM_BP_FAT | GO:0051174~regulation of phosphorus metabolic process | 15 | C5AR1, BLM, SOCS3, CD74, IL31RA, TRIB1, SPRY1, ADRB2, CERKL, CXCR4, DGKD, LAX1, JUN, IL1B, PRKACB | 7.36E-03 |
| SP_PIR_KEYWORDS | disulfide bond | 36 | C3AR1, LTBP1, HLA-DRB1, C3, PRTG, TRGC2, CD74, ITGAM, CXCR4, P2RY2, IGLL1, HEG1, SUCNR1, SRGN, C5AR1, NRXN3, SELL, FBN1, MFGE8, PIK3IP1, EMILIN2, FURIN, SLAMF1, FZD4, PLAUR, SLIT3, CD163, SIGLEC1, CD38, ADRB2, FCAR, HBEGF, TREM1, PTAFR, HLA-DRA, CLEC1B | 9.39E-03 |
| INTERPRO | IPR006209:EGF | 8 | LTBP1, NRXN3, SELL, FBN1, HBEGF, HEG1, MFGE8, SLIT3 | 1.05E-02 |
| GOTERM_BP_FAT | GO:0001775~cell activation | 11 | KLF6, PLSCR1, BLM, CXCR4, LAX1, FLT3, RELB, BCL3, SLAMF1, CD74, ITGAM | 1.20E-02 |
| GOTERM_BP_FAT | GO:0045859~regulation of protein kinase activity | 12 | SPRY1, ADRB2, C5AR1, CERKL, BLM, CXCR4, LAX1, DGKD, IL1B, PRKACB, CD74, TRIB1 | 1.32E-02 |
| GOTERM_BP_FAT | GO:0043549~regulation of kinase activity | 12 | SPRY1, ADRB2, C5AR1, CERKL, BLM, CXCR4, LAX1, DGKD, IL1B, PRKACB, CD74, TRIB1 | 1.34E-02 |
| GOTERM_BP_FAT | GO:0045321~leukocyte activation | 10 | KLF6, BLM, CXCR4, LAX1, FLT3, RELB, BCL3, SLAMF1, CD74, ITGAM | 1.35E-02 |
| GOTERM_BP_FAT | GO:0006952~defense response | 16 | CIITA, C3AR1, KYNU, C5AR1, C3, ANXA1, CD74, IL31RA, CD163, SIGLEC1, CXCR4, BCL3, IL1B, PTAFR, CLEC1B, HLA-DRA | 1.52E-02 |
| GOTERM_BP_FAT | GO:0051338~regulation of transferase activity | 12 | SPRY1, ADRB2, C5AR1, CERKL, BLM, CXCR4, LAX1, DGKD, IL1B, PRKACB, CD74, TRIB1 | 1.55E-02 |
| GOTERM_BP_FAT | GO:0009611~response to wounding | 14 | CIITA, KLF6, C3AR1, C3, F13A1, ANXA1, CD163, PLAUR, PLSCR1, SIGLEC1, CXCR4, HBEGF, IL1B, PTAFR | 2.14E-02 |
| GOTERM_BP_FAT | GO:0042330~taxis | 8 | C3AR1, C5AR1, IL16, CXCR4, IL1B, ITGAM, PTAFR, PLAUR | 2.36E-02 |
| GOTERM_BP_FAT | GO:0006935~chemotaxis | 8 | C3AR1, C5AR1, IL16, CXCR4, IL1B, ITGAM, PTAFR, PLAUR | 2.36E-02 |
| GOTERM_BP_FAT | GO:0042330~taxis | 8 | C3AR1, C5AR1, IL16, CXCR4, IL1B, ITGAM, PTAFR, PLAUR | 2.36E-02 |
| GOTERM_BP_FAT | GO:0006935~chemotaxis | 8 | C3AR1, C5AR1, IL16, CXCR4, IL1B, ITGAM, PTAFR, PLAUR | 2.36E-02 |
| GOTERM_BP_FAT | GO:0006928~cell motion | 13 | ZFAND5, SHROOM2, IL16, CXCR4, BTG1, NRXN3, ARID5B, ANXA1, IL1B, HBEGF, ITGAM, SLIT3, PLAUR | 2.49E-02 |
| SP_PIR_KEYWORDS | membrane | 59 | QPCTL, PRTG, TRGC2, MFSD2A, SLC26A11, IL31RA, SPRY1, SLC2A3, CXCR4, DYNC2H1, FNDC3A, C5AR1, NRXN3, MFGE8, SEL1L3, PIK3IP1, PLAUR, CD163, SIGLEC1, CD38, ADRB2, LAX1, SLC26A9, LRMP, TREM1, PTAFR, HLA-DRA, NEU4, CLEC1B, C3AR1, SHROOM2, HLA-DRB1, SORCS2, CD74, ITGAM, TSC22D3, ACSL1, LPCAT1, DGKD, P2RY2, KCNE1, HEG1, SUCNR1, MS4A4A, SELL, FLT3, ANXA1, FURIN, SLAMF1, FZD4, DOCK4, PLSCR1, FCAR, TMEM163, MBOAT7, HBEGF, GK, JAK3, SYTL3 | 2.85E-02 |
| SP_PIR_KEYWORDS | egf-like domain | 8 | LTBP1, NRXN3, SELL, FBN1, HBEGF, HEG1, MFGE8, SLIT3 | 3.21E-02 |
| GOTERM_BP_FAT | GO:0042110~T cell activation | 7 | BLM, CXCR4, FLT3, RELB, BCL3, CD74, ITGAM | 3.69E-02 |
| GOTERM_BP_FAT | GO:0007243~protein kinase cascade | 11 | C5AR1, CXCR4, SOCS3, LAX1, IL1B, BCL3, JAK3, PRKACB, CD74, IL31RA, TRIB1 | 3.82E-02 |
| UP_SEQ_FEATURE | disulfide bond | 36 | C3AR1, LTBP1, HLA-DRB1, C3, PRTG, TRGC2, CD74, ITGAM, CXCR4, P2RY2, IGLL1, HEG1, SUCNR1, SRGN, C5AR1, NRXN3, SELL, FBN1, MFGE8, PIK3IP1, EMILIN2, FURIN, SLAMF1, FZD4, PLAUR, SLIT3, CD163, SIGLEC1, CD38, ADRB2, FCAR, HBEGF, TREM1, PTAFR, HLA-DRA, CLEC1B | 3.86E-02 |
| GOTERM_BP_FAT | GO:0002684~positive regulation of immune system process | 9 | C3AR1, CD38, BLM, C3, LAX1, IL1B, CACNB4, CD74, HLA-DRA | 3.95E-02 |
| GOTERM_BP_FAT | GO:0002521~leukocyte differentiation | 7 | KLF6, BLM, FLT3, RELB, BCL3, CD74, IL31RA | 3.97E-02 |

**Table S4.** Number of PCR cycles

| **Input amount of total RNA** | **Input amount of cells** | **Typical number of PCR cycles** |
| --- | --- | --- |
| 10ng | 1000 cells | 7-8 |
| 1ng | 100 cells | 10-11 |
| 100pg | 10 cells | 14-15 |
| 10pg | 1 cell | 17-18 |

**SUPPLEMENTARY DATA**

Dataset S1. Loci filtered due to inconsistency between genotype and RNAseq reads

Dataset S2. A summary of mRNA transfer from macrophage to adipocyte

Dataset S3. A summary of mRNA transfer from adipocyte to macrophage

Dataset S4. Differential genes between cell lines cultured alone and co-cultured

**REFERENCES**

1. Garcia NA, Moncayo-Arlandi J, Sepulveda P, Diez-Juan A: **Cardiomyocyte exosomes regulate glycolytic flux in endothelium by direct transfer of GLUT transporters and glycolytic enzymes**. *Cardiovasc Res* 2016, **109**(3):397-408.

2. Thery C, Amigorena S, Raposo G, Clayton A: **Isolation and characterization of exosomes from cell culture supernatants and biological fluids**. *Current protocols in cell biology / editorial board, Juan S Bonifacino [et al ]* 2006, **Chapter 3**:Unit 3.22.

3. Hagen J, te Brinke H, Wanders RJA, Knegt AC, Oussoren E, Hoogeboom AJM, Ruijter GJG, Becker D, Schwab KO, Franke I *et al*: **Genetic basis of alpha-aminoadipic and alpha-ketoadipic aciduria**. *J Inherit Metab Dis* 2015, **38**(5):873-879.

4. Kopylova E, Noe L, Touzet H: **SortMeRNA: fast and accurate filtering of ribosomal RNAs in metatranscriptomic data**. *Bioinformatics (Oxford, England)* 2012, **28**(24):3211-3217.

5. Bolger AM, Lohse M, Usadel B: **Trimmomatic: a flexible trimmer for Illumina sequence data**. *Bioinformatics (Oxford, England)* 2014, **30**(15):2114-2120.

6. Van der Auwera GA, Carneiro MO, Hartl C, Poplin R, Del Angel G, Levy-Moonshine A, Jordan T, Shakir K, Roazen D, Thibault J *et al*: **From FastQ data to high confidence variant calls: the Genome Analysis Toolkit best practices pipeline**. *Current protocols in bioinformatics / editoral board, Andreas D Baxevanis [et al]* 2013, **11**(1110):11 10 11-11 10 33.

7. Li H: **A statistical framework for SNP calling, mutation discovery, association mapping and population genetical parameter estimation from sequencing data**. *Bioinformatics* 2011, **27**(21):2987-2993.

8. Trapnell C, Pachter L, Salzberg SL: **TopHat: discovering splice junctions with RNA-Seq**. *Bioinformatics* 2009, **25**(9):1105-1111.
